# ISAnalytics enables longitudinal and high-throughput clonal tracking studies in hematopoietic stem cell gene therapy applications

**DOI:** 10.1093/bib/bbac551

**Published:** 2022-12-21

**Authors:** Giulia Pais, Giulio Spinozzi, Daniela Cesana, Fabrizio Benedicenti, Alessandra Albertini, Maria Ester Bernardo, Bernhard Gentner, Eugenio Montini, Andrea Calabria

**Affiliations:** IRCCS Ospedale San Raffaele, San Raffaele Telethon Institute for Gene Therapy (SR-Tiget), Milan, Italy; IRCCS Ospedale San Raffaele, San Raffaele Telethon Institute for Gene Therapy (SR-Tiget), Milan, Italy; IRCCS Ospedale San Raffaele, San Raffaele Telethon Institute for Gene Therapy (SR-Tiget), Milan, Italy; IRCCS Ospedale San Raffaele, San Raffaele Telethon Institute for Gene Therapy (SR-Tiget), Milan, Italy; IRCCS Ospedale San Raffaele, San Raffaele Telethon Institute for Gene Therapy (SR-Tiget), Milan, Italy; IRCCS Ospedale San Raffaele, San Raffaele Telethon Institute for Gene Therapy (SR-Tiget), Milan, Italy; IRCCS San Raffaele Scientific Institute, Vita-Salute San Raffaele University, Milan, Italy; IRCCS Ospedale San Raffaele, San Raffaele Telethon Institute for Gene Therapy (SR-Tiget), Milan, Italy; IRCCS Ospedale San Raffaele, San Raffaele Telethon Institute for Gene Therapy (SR-Tiget), Milan, Italy; IRCCS Ospedale San Raffaele, San Raffaele Telethon Institute for Gene Therapy (SR-Tiget), Milan, Italy

**Keywords:** Clonal tracking, Vector integration sites, Gene therapy, Barcoding, Hematopoietic stem cells, Lineage tracking

## Abstract

Longitudinal clonal tracking studies based on high-throughput sequencing technologies supported safety and long-term efficacy and unraveled hematopoietic reconstitution in many gene therapy applications with unprecedented resolution. However, monitoring patients over a decade-long follow-up entails a constant increase of large data volume with the emergence of critical computational challenges, unfortunately not addressed by currently available tools. Here we present ISAnalytics, a new R package for comprehensive and high-throughput clonal tracking studies using vector integration sites as markers of cellular identity. Once identified the clones externally from ISAnalytics and imported in the package, a wide range of implemented functionalities are available to users for assessing the safety and long-term efficacy of the treatment, here described in a clinical trial use case for Hurler disease, and for supporting hematopoietic stem cell biology *in vivo* with longitudinal analysis of clones over time, proliferation and differentiation. ISAnalytics is conceived to be metadata-driven, enabling users to focus on biological questions and hypotheses rather than on computational aspects. ISAnalytics can be fully integrated within laboratory workflows and standard procedures. Moreover, ISAnalytics is designed with efficient and scalable data structures, benchmarked with previous methods, and grants reproducibility and full analytical control through interactive web-reports and a module with Shiny interface. The implemented functionalities are flexible for all viral vector-based clonal tracking applications as well as genetic barcoding or cancer immunotherapies.

## Background

**Figure 1:**
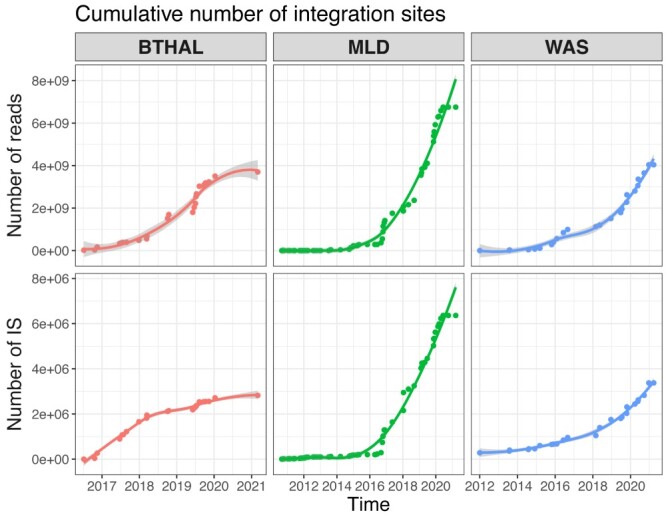
Data accumulation in decade-long clinical trial monitoring. The cumulative number of integration sites retrieved over time for three major clinical trials, BTHAL (in red), MLD (in green) and WAS (in blue). On top, the number of cumulative raw reads retrieved produced after sequencing, and on the bottom the number of cumulative IS retrieved. Each dot is a new sequenced library and the curve is a spline connecting all points (0.8 CI).

Clonal tracking studies are emerging approaches to analyze hematopoietic stem cells (HSC) *in vivo* and characterize hematopoiesis. In HSC gene therapy (GT), clonal tracking studies have been extensively used to assess the safety and efficacy of the treatment [1–10] and dissect stem cell fate and activity [11]. Upon autologous transplantation of corrected cells, the engrafted cells become the new HSC clones that will reconstitute the hematopoietic system of the patient. Each clone is univocally identified by the vector integration site (IS), used as a genetic label, which is stably inherited by all stem cell progeny. For this reason, vector ISs represent the ideal molecular tool for *in vivo* clonal tracking and for uncovering HSC biology [12, 13]. Several molecular technologies are able to retrieve vector IS using custom PCR methods and deep-sequencing approaches of the vector-host genome junctions [14–17]. Each sequencing file is derived from a library of PCRs containing several multiplexed samples, and is then processed by specific bioinformatics tools to identify and quantify IS [18–21] generating a large, sparse matrix for each library. The IS matrix contains all samples in columns, all identified IS in rows, and the value of each IS corresponds to the number of cells or reads retrieved in that sample. In many clinical applications, regulatory authorities require long-term follow-up monitoring, even over decade-long periods, with IS analysis for the assessment of the safety and efficacy of the treatment and for the approval and commercialization of the therapy. Sequencing data volume will thus increase exponentially over the observational time, sustained by the speed-up of technological improvements. Since a comprehensive analysis of all patients with a full clonal tracking matrix requires the integration of all clinical trial data and data scale-up is seldomly approached by a corresponding computational improvement, we rapidly face computational bottlenecks, hardware limitations and analytical stack/overflow (Figure 1). For example, our recent clinical studies for the treatment of metachromatic leukodystrophy (MLD) [2, 22], Wiskott-Aldrich syndrome (WAS) [12] and β-Thalassemia (BTHAL) [23] exploited the most advanced high-throughput sequencing technologies (such as Illumina Nova-seq platforms), which generated up to 500 million reads per single library composed of 150 samples and potentially resulting in ~500 000 IS. Current bioinformatics solutions are unfortunately suffering from computational limitations and new software is required for efficient, scalable and reproducible longitudinal clonal tracking enabling the integration of several clinical studies, thus following several millions of clones in thousands of observations/samplings.

Furthermore, clonal tracking analysts, dealing with large volume data and involved in high-dimensional longitudinal studies, should be focused on biological questions and their readouts. Indeed, the ideal software solution should support the design of a high-level process of data analysis tailored by the biological questions, simplifying data handling and returning easy-to-use reports on both final results and on all intermediate steps throughout the overall analytical workflow/process, granting full control and reproducibility. Despite only few of the available software for IS tracking provide specific functions for *ad hoc* analyses [19, 24], none of them is designed to support end users on a flexible design of their high-level analytical process to answer custom biological questions leveraging on a portfolio of ready-to-use functionalities/tools for data integration, harmonization, filtering and normalization. Moreover, none of the current bioinformatics resources can be fully integrated with a software for IS identification and quantification [18–21] nor with any laboratory information management software (LIMS) [25] to enable a complete workflow management system and support setting up standard operative procedures.

To overcome all the above-mentioned issues, we here present ISAnalytics, a novel R package developed for a comprehensive, reproducible and scalable clonal tracking, designed to leverage on metadata for structuring a full analytical process from sequencing reads to biological questions. The key novelty features of our work are as follows: (1) in terms of computer science, we designed and developed a new metadata-driven approach through which a user can focus only on metadata to run the analyses rather than directly handling data, thus easily supporting scientists to focus on their questions rather than on computational or programmatic aspects; (2) ISAnalytics realizes a full data integration with pre-processing tools and it is generalized to support every integration in custom laboratory workflows, thus helping on standardizing laboratory procedures; (3) on the computational side, we used efficient data structures avoiding sparse data and developing all functions with parallel processing; (4) on the biological side, ISAnalytics allows full implementation of clonal tracking features supporting reproducible data analysis and full control with web reports and a Shiny interface.

## Results

In this section, we will describe the package design and development with benchmarks on the performances. Relevant functions for clonal tracking studies are described with examples and detailed in methods. To provide a real case application, we will present all results applied to the recently published study related to the lentiviral vector (LV) HSC-GT clinical trial for Hurler syndrome (mucopolysaccharidosis type I, Hurler variant—MPSIH) [8] as well to simulated data.

### Metadata-driven approach and software integration

**Figure 2:**
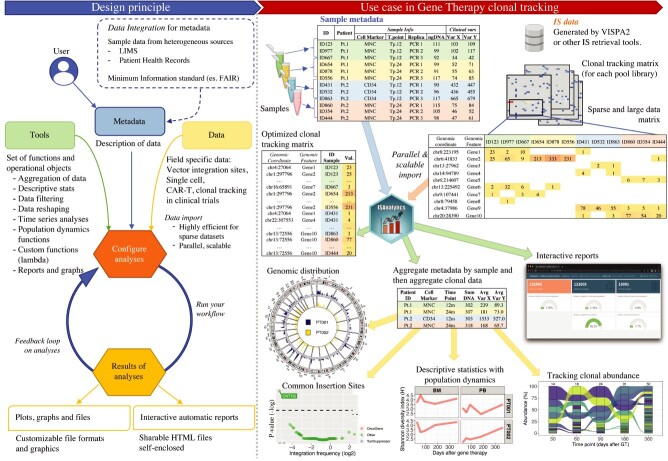
ISAnalytics design principle and real case application example. The design principle of ISAnalytics is based on a full integration of data with their descriptors (metadata). Users access metadata to configure their analyses (in orange) based on data (in light yellow) and use pre-configured sets of functions or custom functions (in light green). Then the process is run, and the users access results of the analyses both with graphical objects (graphs, etc.) and interactive reports. On the right, an example of application of a typical workflow: sample metadata and IS data, in the form of sparse matrices, are imported exploiting parallel computing approaches to reduce processing time and memory footprint. After reshaping data in a more computationally efficient format, the workflow can proceed with data aggregation, which allows the union of PCR replicates referring to the same biological sample, and users can then decide in autonomy which biological questions they wish to answer through the wide range of analyses functions provided by the package, which not only provide the actual data but also a convenient and fast way to visualize result with appropriate plotting functions and interactive dashboards.

ISAnalytics is designed with a metadata-driven approach, in which data descriptors are the key entry for all analytical processes (Figure 2), and clonal data are linked to metadata by a key field. Samples are usually recorded with specific information related to their origin (e.g. patient ID or mouse ID, tissue, time point, cell marker, etc.) and the experimental procedures in place to retrieve clones (PCR method, DNA amount, etc.), and patient’s data (disease, treatment age, conditioning, etc.). These data can be summarized with minimum information standards following the FAIR approach (https://www.go-fair.org/fair-principles/) and archived in digital lab-books or LIMS, such as adLIMS [25]. In particular, ISAnalytics acquires a metadata file in simple text with arbitrary data fields summarizing the input samples to analyze (import details in Methods section) with the unique mandatory field related to sample identifiers that must be identical to the clonal data column headers. Most of the LIMS is provided with exporting procedures to extract sample metadata; indeed, the metadata file can be easily generated by LIMS solutions, or by custom procedures if data are stored in other formats (from relational databases to simple spreadsheets or flat files) (see Supplementary Material for details). Clonal data are usually returned as sparse data matrix by many current bioinformatics tools, as previously mentioned, and each sample is referenced by a column of the data matrix. Storing in the header of the column of the data matrix the same ID reported in the metadata key field allows a direct connection between data and metadata. VISPA2 [18], for example, as well as INSPIIRED [19] or GENE-IS [21], generates a single large and sparse data matrix for each single sequencing library that multiplexed hundreds of samples. We designed ISAnalytics to manage, process, analyze and query clonal data driven by metadata such that end users would need only to customize their analytical processes focusing on metadata and not on large (clonal) data and their computational complexity of management. Our tool acquires a general sparse matrix of clonal data returned by IS identification software and can be fully embedded downstream a laboratory workflow of IS analysis in which samples are managed and tracked by an LIMS (Supplementary Figure S1).

### Clonal tracking features and available functionalities

**Figure 3:**
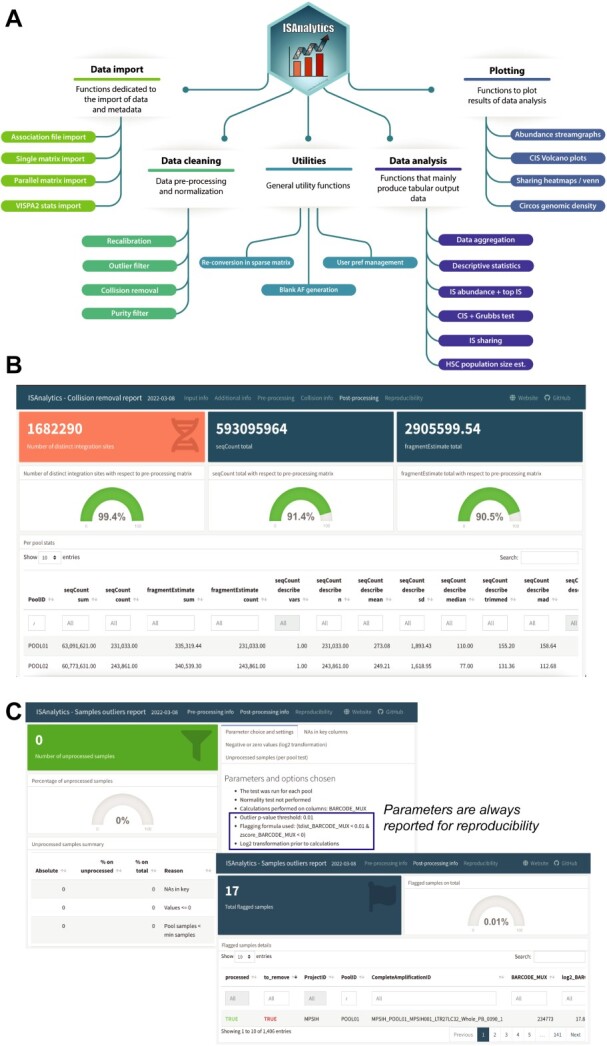
Summary functionalities and sample reports. (**A**) Mind map illustrating the main functionalities of the package divided in five thematic areas. Data import: group of functions dedicated to the import of tabular files containing data and metadata from disk; data cleaning: contains all functions dedicated to data cleaning and pre-processing; utilities: general purpose functions, useful for common operations but not specific for any analytical workflow; data analysis: core of the package, contains the actual analysis functions that produce results for answering biological questions; plotting: easy to use functions to plot results of analyses functions. (**B**) and (**C**) show examples of interactive reports obtained from the workflow on MPSIH data. (B) Report of the collision removal step with the post-processing summary tab. The interactive reports visualize a summary of the entire procedure with the total number of distinct IS after processing, the total amount of the quantifications after collisions removal both in absolute numbers and in percentages relative to the pre-processed matrix in input, followed by a series of descriptive statistics for each pool. (C) Report of the outlier filtering procedure showing the actual number of flagged samples with the associated details and all calculations performed.

Clonal tracking studies require both standard analyses and new custom features. ISAnalytics implemented many useful features that allow end users to design their own analytical processes (Figure 3A). Here we present the most useful functions for the analysis of IS in clinical trials that supported safety, long-term efficacy and basic biology research. Additional features, functions and software design details are available in the online documentation and in Supplementary Material (Supplementary Figures S2 and S3).

#### Data import, filtering and pre-processing

When integrated with VISPA2, IS matrices can be imported guided by the metadata file: only the samples in the metadata file will be imported, allowing full control of the analyses. ISAnalytics acquires the paths of data files listed in the appropriate metadata field and imports them, summarizing all resulting steps with an interactive plot. The interactive plot supports users in finding potential problems during the operation of import of each pool and allows a graphical inspection of all the imported data.

Despite a relatively short follow-up of 18 months after GT of MPSIH patients, a large amount of clonal tracking data was collected: 11 sequencing libraries (pools) generated and sequenced with the latest Illumina Nova-seq technology (Supplementary Figure S3A and B). The number of raw reads was >2 billion corresponding to an overall number of >1.6 million clones tracked across 1406 samples (composed by time and lineages). Due to the large volume of data for each pool, the MPSIH dataset presented critical computational issues due to data accumulation. We tested and benchmarked ISAnalytics by importing all data at the same time and performing incremental data import based on the time of collection, thus replicating the scenario of updating all clonal tracking data every 6 months. To perform analyses of incremental size, we used groups of sequencing libraries composed by 3, 7 and 11 datasets. We then parsed the logs to summarize the computational costs (memory peak and running time). Moreover, the import function has been tested using ISAnalytics, with parallel import configured with four processes, and compared with the canonical data frame import of sparse matrix, configured with one process (Supplementary Figure S3C). Our results showed that ISAnalytics import resulted more efficiently (10 to >10^3^ times) in terms of computation time with a clear improvement using larger datasets composed by 11 sequencing libraries (Supplementary Figure S3D). Even better performance has been observed in terms of memory peak (Supplementary Figure S3E) with a minimum improvement of 10^7^ (range 10^7.31^, 10^8.99^) (Supplementary Figure S3F). In terms of data exploration, we used interactive plots to identify any potential issues (Supplementary Figure S4A and B).

Once IS data are imported, users can perform raw data quality analysis and filtering (see Methods section for details). We implemented three main functionalities: (1) IS recalibration, to avoid wobbling of up to three base pairs around the IS that could impact in missing IS tracking in different sequencing pools; (2) collision removal, to remove any potential cross-contaminations among samples that may eventually lead to assign the same IS to distinct independent patients; and (3) filtering of low-quality samples, to remove low-quality PCR samples (potentially only one of the sample replicates) in terms of the expected number of sequencing reads. Further implementation details are described in Supplementary Material, accompanied by Supplementary Figures S5–S7. In the MPSIH clinical trial, we obtained 1 692 420 IS by VISPA2 with a total number of 648 687 652 mapped reads including all samples and pools, considering that the recalibration step combined 2.5% of the neighboring IS within ±3 bp. The total number of IS identified as collisions was 131 545, corresponding to 7.77% of the overall IS. With the method of collision identification and removal, we have been able to reassign 121 415 IS (>92% of the collisions) to the source patient, thus drastically reducing the number of discarded IS by contaminations to only 10 130 (0.6% of the total IS). The final number of IS was then 1 682 290 clones, with a total number of 593 095 964 reads of IS (Figure 3B). We then filtered MPSIH PCR samples by the number of sequencing reads (see Supplementary Material) and we obtained that 17 elements out of 1406 samples resulted undersequenced and needed to be filtered out. ISAnalytics provides a Shiny web application, ‘NGSdataExplorer’ (Supplementary Figure S8, Supplementary Material), to explore descriptive statistics data of raw sequencing data with a visual and intuitive interface, useful for changing the default parameters of data filtering.

#### Data aggregation

Data aggregation is a pivotal function in ISAnalytics since it allows to combine IS data of different samples in a new aggregated column exploiting the metadata file. For example, a single sample may present several technical replicates, as many recent studies generate [8, 15, 16], that users need to collate in a single data as the union of the replicates with the quantification reported as the sum of the observed values. Indeed, even if a clone has been observed in only one of the replicates, the aggregated representation will present that clone and hide the source PCR, and the assigned value will be derived only by the single replicate. Moreover, clonal tracking studies may need to aggregate IS data at different levels of granularity, from single samples at a specific time point, to the level of tissues combining all time points, or at the patient level. Based on these requirements, we designed the aggregation function using the metadata file, since metadata is able to describe the dependencies and relationships among the samples at a single PCR resolution. Indeed, we developed a lambda function that acquires IS data, metadata and an aggregation function(s) (default is the sum of values), even user-defined functions (see Methods, Supplementary Material). Data aggregation has been essential for MPISH patients since we used this function (1) to combine all replicates together, thus obtaining a unique sample clonal dataset; (2) to analyze the clonal repertoire over time and lineages (aggregating by time and lineage) in terms of clonal population diversity and abundance assessment; and (3) to perform patient aggregation for genomic distribution profiles and analyses.

#### Genomic distribution of IS

**Figure 4:**
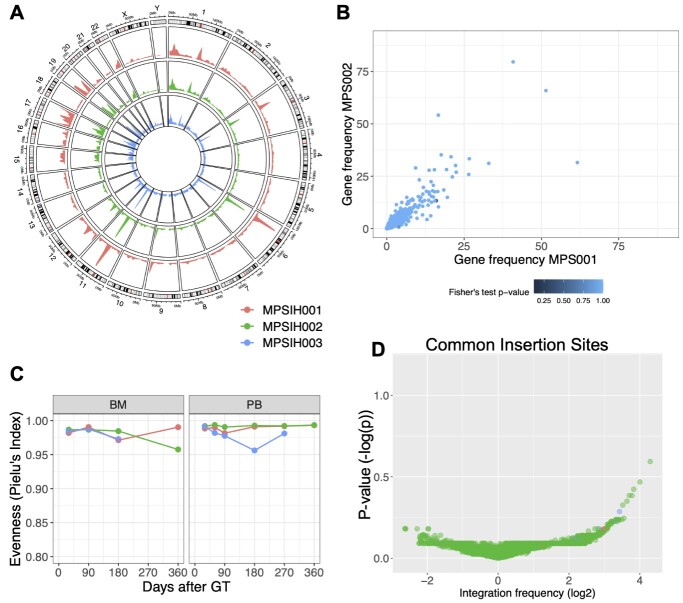
Clonal tracking in MPSIH patients. (**A**) Plotted results of the analysis workflow for MPSIH clinical trial data. (A) Circos plot representation of genomic distribution IS for the first three patients MPSIH001 (in red), MPSIH002 (in green) and MPSIH003 (in blue). (**B**) Scatter plot of the comparison between genomic tracks between patients 1 and 2. Each data point represents a gene that has coordinate the gene frequency of patient 1 (on *x* axis) and the gene frequency of patient 2 (on *y* axis). Points are colored according to their *P*-value for the Fisher’s exact test and are eventually highlighted and labeled in red if the *P*-value is lower than the set significance threshold for the test (by default 0.05); here no genes resulted significantly different. (**C**) Clonal population evenness (Pielu’s index, *y*-axis) over time (*x*-axis) retrieved in whole bone marrow (BM) and peripheral blood (PB). (**D**) Volcano plot of the results for CIS-Grubbs test, representing on *x*-axis the gene integration frequency (expressed in log2) and on *y*-axis the average *P*-value resulted by the Grubbs test (−log *P*-value) among the first three patients.

To observe the targeted regions by the vector, ISAnalytics provides a function to plot the genomic distribution of IS represented as circos plot (Figure 4A). Since the genomic representation is purely qualitative, we also developed and released a function to compare two genomic tracks gene by gene and report gene-based *P*-values (performing the Fisher exact test between the two samples with the contingency table reporting the number of IS observed annotated for each gene). This function could be useful to compare two independent patients to inspect for patient-specific integration bias or between two distinct vectors to compare the genomic preference of vector integration (Figure 4B). As expected, no statistical differences were observed when comparing two lentiviral integration profiles, since vector integration is mainly driven by biological factors (such as the recombinant human protein LEDGF [26, 27]) rather than patient properties.

#### Clonal population complexity and descriptive statistics

We developed a lambda function to process descriptive statistics, both with common functions (e.g. variance, mean, standard error, etc.) and custom/user-defined functions. In the latter case, we embedded several indexes of population diversity such as the Shannon-Weaver index, Simpson, inverse Simpson, Rènyi index and evenness indexes (for equitability) such as Pielou. Population diversity and evenness indexes are widely used in GT applications to assess the safety and long-term efficacy of the treatment. In particular, in a highly diverse cell population, the Shannon index will return high values. On the contrary, in the cases of clonal expansions or treatment exhaustion the Shannon index will return lower values. When analyzing Pielou index ranging from 0 to 1, the highest is the score, the more polyclonal is the population. Indeed, by analyzing the results of the Shannon diversity index over time, users quantify the clonal complexity in all cell populations and understand globally the dynamics of cell composition; similarly if analyzing the Pielou evenness index, as in the case of MPSIH (Figure 4C). Given that the clonal population complexity in the MPSIH patients was observed >0.95 of the Pielu index (measuring the evenness of the clones), we could confirm that no oligoclonal patterns were detected.

#### Common insertion sites

Common insertion sites (CIS) genes are genomic regions significantly targeted by vectors compared to other genes, and CIS are often used to highlight events of genotoxicity [28]. For this reason, CIS genes are always required when analyzing the biosafety of the treatment, both in preclinical and in clinical applications [6]. Several analytical methods have been developed to quantify CIS genes [28–30]. We developed in ISAnalytics the approach based on Grubbs test for outliers [28] and released the results as volcano plot. Combining the results of MPSIH patients (Figure 4D), we observed that no genes resulted significantly overtargeted by LV integration (alpha error at 0.5).

#### Clonal abundance over time

**Figure 5:**
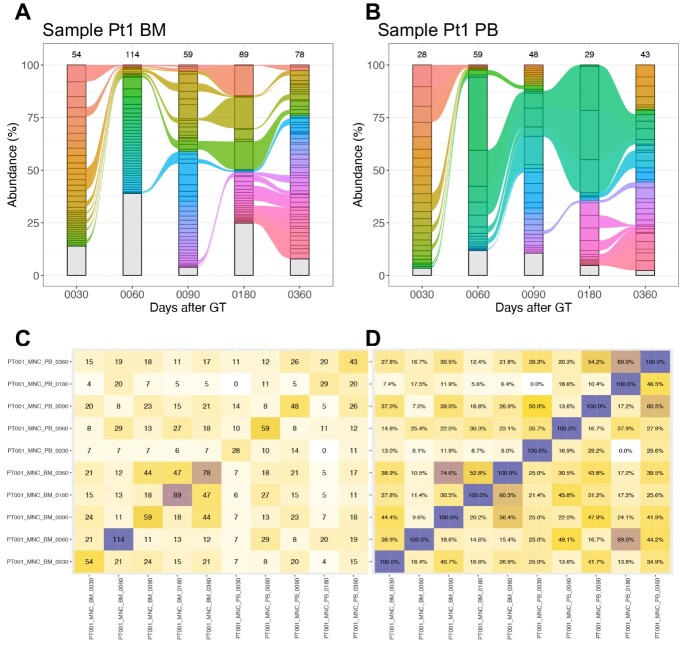
Streamgraphs for clonal abundance and IS sharing. (**A**, **B**) Streamgraphs of the clonal abundance calculated from the package included sample data, both refer to MNC cell populations in patient 1 (Pt1) in two different tissues (bone marrow—BM in A, peripheral blood—PB in B). Colored ribbons indicate a relative clonal abundance >1% in at least one time point, all IS below this threshold are grouped together in the gray strata. (**C**) Absolute number of distinct IS shared between samples visualized as colored heatmap (square matrix). In the example, each row identifies a sample (named with patient id, cell marker, tissue and time point in days). Within each cell is reported the number of shared IS expressed as absolute number (C) or relative percentage (**D**).

Clonal abundance is an important criterion for quantifying potential clonal expansion events in the assessment of safety. The size of a clone is proportional to the number of observed cells harboring the same IS. If the imported data matrix contained the quantification of each IS reported as the number of observed or estimated genomes, as computed by the R package SonicLenght [31], the abundance of each IS can be calculated as the relative fraction of cells of that IS on the total number of cells (sum over all IS) for each sample. Tracking the abundance of each clone over time and samples is important to quantify any expansion. For this reason, in ISAnalytics we implemented a function to compute the clonal abundance and to plot the results in stream graphs (Figure 5A and B) in which each time point shows a stacked bar plot with clones that are colored by a unique color if their abundance overcomes a threshold (1% in the example) in at least one time point and connected by a ribbon if recaptured in adjacent time points. The abundance plots of MPSIH patients were already presented [8]; indeed, here we show a general case of a simulated dataset.

#### Clonal tracking by IS sharing among samples

In many biological analyses, clonal tracking methods require following IS across samples, time points and lineages. Tracking IS among samples is usually approached by set operations, typically based on the number of shared IS between pairs of samples and then returning the relative percentage on the union of the two sets or on each of the two independent sets. For this reason, we implemented in ISAnalytics a function to calculate the number of shared IS among input samples and to return both absolute numbers of shared IS and relative percentages. From these results, ISAnalytics can support generating heatmaps and saving sharing results for other analyses. To report examples of the functionality, we used a sample dataset embedded with ISAnalytics reporting two patients for which MNC samples were collected and IS tracked over time and tissues. Computing the sharing analysis by patient with ISAnalytics, we obtained a squared data matrix containing the number of IS observed between pairs of samples (or the relative percentage) and plotted as a heatmap (Figure 5C and D, Supplementary Figure S9, Supplementary Material). This function is useful to calculate the output of CD34 BM cells towards mature cell lineages, as reported in several studies [1, 2, 12, 13].

#### Population size estimate

Capture and recapture statistics have been widely used in ecology to estimate the size of a population in an ecosystem [32]. Several GT studies reused the same statistical models developed in the field of ecology to study the transplanted clonal populations [33], in particular to estimate the size of engrafted and active HSCs that are reconstituting the hematopoietic system of patients [1, 2, 9]. ISAnalytics provides a function to compute the HSC clonal size estimate, both overall, thus including all time points, and over time, thus evaluating triplets of neighboring time points and computing the index as sliding window from the first time point to the last one thus highlighting how the pool size of HSPCs is changing over time. Initial contractions followed by a stabilization have been usually reported as evidence of the exhaustion of short-live committed progenitors leaving the homeostasis to the more primitive HSC. On the other hand, a contraction in the size of HSPC long term may be a mirror of the loss of engraftment and reduced efficacy. We included an example derived from our simulated dataset (Supplementary Figure S10), reported without any claims on the biological interpretation but rather used for software testing.

### Comparative analysis of the tools

Here we reported the comparative analysis of the features with barcodeTrackR, the other publicly available tool for clonal tracking (Table 1, Supplementary Material, Extended Data 1). While barcodeTrackR offers similar functionalities for clonal tracking studies with respect to ISAnalytics, it does not provide full support for the analytical workflow including data import and data pre-processing phases, which are therefore delegated to the user. Moreover, barcodeTrackR uses standard sparse-like data structures (SummarizedExperiment) while ISAnalytics relies on tidy data structures (tibble and data.table). Functionality-wise barcodeTrackR allows the production of plots for samples correlation and a unique set of functions dedicated to clonal bias analyses and distance/similarity between samples, which are not included in ISAnalytics; it also provides a Shiny web interface while ISAnalytics relies on static interactive dashboards for reproducibility purposes.

**Table 1:** Comparison of the functionalities among ISAnalytics and state of the art methods. The comparison highlights pros and cons of every package, divided by macro-areas (leftmost column) and specific functionality (second column on the left)

	**Functionality**	**ISAnalytics**	**barcodetrackR**
**Software**	**Release**	R package published on Bioconductor	R package published on Bioconductor
	**Metadata and association files**	Yes (extensive use, plus hypothesis driven approach)	Yes (embedded in SummarizedExperiment data structure)
	**Maintenance**	Yes (last commit 2022)	Yes (last commit 2021)
**Data import and pre-processing**	**Data import**	Reads files, converts to tidy, additional operations. Supports automatic import of multiple files in parallel.	File reading external to package (base R), then conversion to SummarizedExperiment (sparse). No parallelization (one file at time).
	**Sequencing data filtering**	Yes (by pool with stat)	No
	**Data recalibration**	Yes (with report)	No
	**Collision detection and removal**	Yes (with report)	No
	**Abundance filtering**	Yes	Yes
	**Purity filtering**	Yes	No
**Data Manipulation**	**Data aggregation**	Yes (customizable and metadata driven)	No
**Statistics**	**Descriptive statistics**	Yes (plus custom with lambda functions)	Yes (only specific stats: diversity indexes included are the same as ISAnalytics)
**Clonal Tracking**	**Abundance**	Yes	Yes
	**Samples correlations and similarity**	No	Yes
	**Tracking of top *n* clones in time**	Yes	Yes (plus heatmaps)
	**Clonal distribution over time**	Yes (alluvial plots - not restricted to plot over time)	Yes (area plots - restricted to use time points)
	**Barcode presence in heatmap**	No	Yes
	**Lineage bias analysis**	Yes (using sharing functions)	Yes
	**Tracking unique clones with heatmap** **(either barcodes or ISs)**	No (data available, to plot with simple heatmap call)	Yes
	**Choord diagram**	No	Yes
	**CIS statistics**	Yes (and with plots)	No
	**Clonal sharing and waves of clones over time**	Yes	No
	**HSPC population size estimate**	Yes	No
	**Circos genomic density**	Yes	No
**Reproducibility and human interaction**	**Interactive reports**	Yes	No
	**Shiny interface**	Yes	Yes

## Discussion

Translational science in GT is increasingly demanding for clonal tracking tools, requiring open-source, scalable and maintained software. Here we presented our new software, ISAnalytics, a new open-source tool for clonal tracking with large volume genomics data, such as whole clinical studies with millions of clones to track over time and lineages as shown for the LV HSC GT trial for MPSIH. Our solution is particularly useful in the case of incremental data acquired to monitor the clones of all patients over decades-long periods of time since technological advances in high-throughput deep-sequencing platforms require significant amounts of computational resources, both hardware and software. To overcome this issue, ISAnalytics specifically relies on parallel processing (*divide et impera* approach) and uses optimized data structures for efficient data science methods in R (tidy). Indeed, ISAnalytics showed highly scalable and versatile in analyzing large volumes of clonal tracking data of several patients in different clinical trials, already used in recent GT applications for molecular monitoring of MPSIH patients [8] and in analyzing results of a new experimental technology [15] called LiBIS-seq (liquid biopsy integration site sequencing). Several alternative approaches could have been used for the basic data structure beyond tidy, such as sparseMatrix (R package Matrix), compressed sparse row or compressed row storage matrix (implemented in various R packages such as MatrixExtra or nalgebra_sparse). We decided to leverage on tidy data because of its improved implementation in data science and machine learning modeling, one of the next direction of clonal tracking studies approaching personalized and predictive medicine. Other promising solutions may be related to graph representation of clonal data, in line with the most recent approaches in bioinformatics for considering personal genomic variability.

ISAnalytics provides many functions commonly used for GT applications which are flexible to customizations on user needs. Nevertheless, ISAnalytics is an open-source tool under continuous development, in particular regarding the implementation of new features for basic and translational research, such as statistical analyses of lineage tracking, lineage skewing and clonal dynamics, currently in progress.

We developed ISAnalytics in R and within Bioconductor to be fully compliant with the bio-oriented scientific community and to follow standard software engineering procedures for implementation and maintenance. Despite ISAnalytics is not yet integrating annotation features thus bridging the genomics universe present in R for comprehensive data analysis, we already tested the iRange conversion of the genomic coordinates to fully connect ISAnalytics with the vast majority of the genomic packages and allow users to extend their analyses. On the other hand, other tools may integrate or use ISAnalytics and its features.

To our knowledge, ISAnalytics is the first data science software in biomedical research entirely designed to be metadata-driven. This key feature opens new opportunities to end users to design their own analytical workflows. For example, in a clinical trial ISAnalytics allows aggregating data by different levels of granularity (at sample level, at tissue level, up to patient level), and projecting results over time, supporting hypothesis-driven analyses.

Since ISAnalytics is dedicated to clonal tracking analyses without performing sequencing reads alignments nor IS identification, in terms of data integrations and operations ISAnalytics can be used standalone or embedded in an integrated laboratory workflow, downstream to a LIMS, that supports users in tracking experimental samples and IS retrieval, and to a software for IS identification. We designed ISAnalytics to be fully ready to use combined with VISPA2, supporting a direct analysis of IS data with automated reporting. Moreover, ISAnalytics supports interactive reports for full control of the analytical process and data, granting reproducibility and transparent science. ISAnalytics can be applied in a wide range of clonal tracking applications, from GT with integrating vectors to recombinant adeno-associated vectors GT in liver [34, 35] or other organs, to cancer immunotherapies (i.e., chimeric antigen receptor T cells—CAR-T studies [36]) or barcoding in gene editing [37, 38] (Extended Data 2).

The philosophy behind the package is to provide a complete tool with a good balance between generalization and standardization. Metadata has a central role in the concretization of these concepts, as it allows for differentiated and generalized workflows depending on the content of specific fields, thus making ISAnalytics a suitable primer or template for other analyses/studies that may find their application in biological research and nonbiological fields (such as economics or data science).

## Methods

### Package included functions

#### Data import and file format

ISanalytics imports IS data (IS matrix) with the following file format: in rows, the ISs annotated with genomics labels such as chromosome, integration locus and strand; in columns, besides the first columns reserved to the IS genomic annotations, the different observations (samples) with the column names as identifiers of each sample (no repetitions allowed). Each data cell contains the quantification of the specific IS observed at the specific sample, such as the number of sequencing reads or fragment estimate.

The metadata file contains data and details of the observed samples. The list and content of the columns are free except for the one column containing the identifier of the samples that must correspond to the identifier reported as the column header in the IS matrix file. Additional details and examples of the file format are present in Supplementary Material, Extended Data 2.

#### Data cleaning and pre-processing

##### Recalibration

We define an integration event as a triple:

$$ \mathrm{integration}\ \mathrm{event}=\left(\mathrm{chr},\mathrm{integration}\ \mathrm{locus},\mathrm{strand}\right) $$

Thus, we define the concept of distance between two integration events with the same ‘chr’ and ‘strand’ components as:

$$ D:\left(x,y\right)\in{\mathbb{N}}^2\to \left|x-y\right|\in \mathbb{N} $$

where *x* and *y* represent the corresponding values of the component ‘integration locus’ for the two events.

We label two integration events as ‘near’ if their distance is less than a threshold value, which by default is set to 4 bp.

$$ x\ \mathrm{is}\ \mathrm{near}\ \mathrm{to}\ y\Longleftrightarrow D\left(x,y\right)<\mathrm{threshold} $$

An integration matrix is a set of distinct integrations, the PCR replicate id and the value of the quantification(s): distinct values for these fields map to different rows in the table.

The matrix is scanned with a sliding window approach using a dynamic window size, and integration events that are sufficiently near to each other are condensed into a single event: such event has as a value for the quantification(s) that is the sum of the quantification(s) of the original integration events (Supplementary Figure S5). For reproducibility purposes, the function supports the production of a recalibration map, in the form of a tabular file, which simply contains a mapping between every old integration event and the new corresponding event. Moreover, the function offers more flexibility in considering an integration event strand-specific or not: by default, two integration events with the same value for the fields ‘chr’ and ‘integration locus’ are considered distinct if they have a different value for the ‘strand’ field. Changing this parameter allows processing IS in which the strand information is not available or not relevant. Further details are given in Supplementary Material.

##### Collision removal

A collision event is a clone retrieved in more than one independent sample, such as distinct patients. We consider this event very unlikely given the probability of two independent vectors to integrate in the same genomic position (in base pairs) from 3 × 10^8^ possibilities (the size of the human genome is ~3 × 10^9^, and viral vectors tend to integrate in less than 10% of the genome). Detailed description of the algorithm is reported in Supplementary Method and Supplementary Figure S6. This procedure generates an interactive report on user request, which allows a first quick glance at data before proceeding with further analyses.

##### Identification and removal of sample outliers

Next-generation sequencing (NGS) sequencing of a sample library could return unbalanced numbers of reads per sample. To remove samples with under-represented number of reads that may reflect in an altered number of clones and abundances, ISAnalytics provides a specific function called” outlier_filter”. Since ISAnalytics is downstream of the tools for IS retrieval (that manage raw data, FastQ files), users need to provide ISAnalytics with a file containing the number of raw reads per sample, as returned by VISPA2 statistics. The statistical test used to identify outliers is based on a lambda function (default function is ‘outliers_by_pool_fragments’), and ISAnalytics returns to users the same input data with a flag column showing if the sample resulted as an outlier by any of the tests. For reproducibility purposes, the operation produces an interactive report in the form of a dashboard if the user requests it. The pseudocode of the function with the algorithm design is described in Supplementary Method and Supplementary Figure S7.

##### Purity filter

To exclude potential contaminations within dependent samples (e.g. samples extracted from the same patient) and to consider only reliable clones in specific analyses, we need to filter IS with very low abundance in single samples, as reported in previous studies [1, 2], using lineage information.

As a preliminary step, a filtering operation on the quantification is performed: only rows with a quantification value greater or equal to the minimum input specified value will be kept. After that, ISs to process are identified: only integration events belonging to groups of interest that are shared in at least two groups are flagged to process, while other IS events that do not match these conditions are kept as they are. Subsequently, the real filtering process takes place: for each flagged integration event, the maximum quantification value is identified and used to calculate the ratio $\frac{\max\ \mathrm{value}}{{\mathrm{value}}_i\ }$ for each group of interest *i* – if this ratio is greater than a user defined threshold (by default 10), the clone is discarded.

#### Data aggregation

The package provides a general tool for aggregating (1) clonal data and (2) metadata by grouping data by specific fields. In the clonal matrix, data aggregation combines samples by their common metadata, for example by grouping (union) the PCR replicates of a single sample. Indeed, data aggregation is deeply important in the context of clonal tracking since it enables zooming in and out crossing the different levels of details related to a sample that may change according to each different analysis, from the single PCR to tissue level up to whole patient, or simply removing the temporal dimension and flattening data as a whole. Data aggregation is conceived as the ‘group by’ operation in SQL which then reshapes numeric values with user-defined functions (lambda). The logic is here summarized by the following SQL query with the sum operation as minimal example:

SELECT chr, integration_locus, strand, GeneName, GeneStrand, sample_key, SUM(seqCount), SUM(fragmentEstimate)

FROM (SELECT *

FROM IS_matrix

LEFT JOIN metadata

ON PCR_identifier)

GROUP BY sample_key

Where ‘sample_key’ corresponds to one or more fields that annotate each sample and ‘PCR_identifier’ is the identifier of the single PCR replicates (usually the header name of each observation reported in the clonal matrix in column with its unique key identifier, as described in the import section); seqCount and fragmentEstimate are two available quantifications, by number of reads or estimated fragments, respectively. Our implementation allows high flexibility in terms of input fields and operations (sum, mean, etc.). ISAnalytics implemented data aggregation in the clonal tracking data matrix with the function ‘aggregate_values_by_key’ where the key is a list of fields to be used as superkey in the aggregation to identify unique output groups, and one or more functions to aggregate values (the sum operation is the default selection). The corresponding metadata counterpart, ‘aggregate_metadata,’ solely takes in input the metadata file, the aggregation key and a list of aggregating functions to be applied (defaults are provided in ‘default_meta_agg’).

#### Descriptive statistics

ISAnalytics provides a simple and flexible way to compute different kinds of descriptive statistics simultaneously on multiple numeric columns: even though the user has complete freedom of specifying which functions and statistics he would like to perform by simply following directions in the documentation, defaults are provided and include the count of values, sum of values, mean, median, standard deviation, skew, kurtosis (and other statistics as output of the *describe* function from the R package ‘psych’), Shannon diversity index and the Simpson diversity index. If the input contains genomic coordinates, it is also possible to request a count of distinct integration sites (IS) for each group.

### Common insertion sites and Grubbs test

Common insertion sites are computed and annotated following the rationale proposed by Biffi *et al.* [28] using Grubbs test for outliers.

Briefly, the function computes different calculations for each gene, starting with the integration frequency normalized by the gene length in base pairs. This value is then scaled and corrected, and finally Grubbs test is performed to assign a *P*-value with false discovery rate (FDR) correction. Genes significantly targeted (*P*-value <0.05) are reported in the final output table and in the volcano plot (the latter produced by the by using the function CIS_volcano_plot).

### Integration sites sharing

Given an integration matrix and one or more group identifiers, that is a combination of metadata fields uniquely identifying a group, we can compute the number of distinct IS that are shared between the groups in terms of absolute number and in percentages, more precisely the operation is the equivalent to the intersection of two or more sets. Despite the concept behind it being fairly simple, the function is implemented to offer great flexibility: users can provide in input one or more integration matrices, different group keys for each data frame in input, decide to compute all possible permutations of the comparisons or just distinct combinations for the sake of efficiency (internally done with the support of the package ‘gtools’), keep or drop the actual coordinates of the IS shared for each comparison and finally, since each row can be plotted as a Venn or Euler plot, choose to compute for each row a truth table, which can be used as the input to the function sharing_venn, that internally uses the functions provided by the package ‘eulerr’ to produce the plots. Although the absence of a specific limitation for the number of sets involved in each intersection, it is important to note that Venn plots have a maximum limit of five sets. Heatmaps offer an alternative visual representation of sharing data with the function sharing_heatmap: for this kind of plot, it is necessary to have only two sets in the comparison and a better visualization is offered when all permutations are computed (Figure 5C and D).

A further application of IS sharing is offered by the function iss_source, which aims to track the single shared IS from its first observed time point. Results can be easily plotted as a stacked bar plot to better visualize the contribution of IS events of previous time points on each point in time observed (Supplementary Figure S9). The logic behind the function only slightly differs from simple sharing: the reference table, given by the user as input, is pre-processed in a way that IS events that occur in more than one timepoint are retained and labeled only with the first timepoint they were observed in. Only afterwards a step of IS sharing through the function is_sharing is completed, to provide the final output of the function.

### Estimate of the HSCs population size

The function ‘HSC_population_size_estimate’ returns the estimated number of the clonal population, for example the estimated size of HSPCs, through capture-recapture models (Chao1) using both closed population and open population models. Estimates are computed by the package ‘Rcapture.’ The user is free to specify the populations on which the models are applied through the appropriate function arguments, along with additional tuning parameters such as thresholds for preliminary filtering on different quantifications, a mapping between cell markers and cell lineages information and one or more stable time points. Results can be plotted easily with the function HSC_population_plot (Supplementary Figure S10).

## Authors’ Contributions

G.P. co-designed and developed all code and benchmarks. G.S. supported the prototype analysis and VISPA2 aggregation. F.B. and A.A. performed sample processing and analyzed raw sequencing data, testing the Shiny interface. D.C. performed end user testing on clinical data and other in vivo clonal tracking datasets. B.G. and M.E.B. provided clinical sample material and reviewed the manuscript. E.M. supervised the project in all clonal functionalities and supported data interpretation. A.C. conceived and supervised the project, interpreted data, wrote the manuscript and coordinated the work. All authors contributed to the manuscript.

Description of the Organization

The mission of *San Raffaele Telethon Institute for Gene Therapy* (SR-Tiget) is to perform cutting-edge research in gene and cell therapy and to translate its results into therapeutic advances, focusing on genetic diseases with the characterization of biological properties and physiopathological processes, the design and optimization of safety and efficacy of novel gene and cell therapy platforms, and their development from preclinical models to first-in-human testing.

**Key Points**

- *ISAnalytics* implements many useful functions for the assessment of the safety and efficacy in gene therapy applications (required by regulatory authorities) as well as for basic scientific questions on clonal dynamics and lineage tracking.
- *ISAnalytics* supports reproducible data analysis and full control of results with web reports and through a Shiny interface.
- *ISAnalytics* implements a metadata-driven approach through which users focus only on data descriptors to design and run their analyses rather than data handling, such that scientists will be focused on their biological questions rather than on computational or programmatic aspects.
- *ISAnalytics* can be embedded within a laboratory workflow since it has been developed to be fully integrated with laboratory information management systems (which usually manage sample metadata) and bioinformatic tools for clonal identification, thus supporting the standardization and automation of laboratory procedures.
- On the computational side, *ISAnalytics* is implemented with efficient and optimized data structures and it is fully parallelized; moreover, it is released as open-source Bioconductor package, with Github integration and under continuous testing using TravisCI and GitHub actions that perform automated checks on all operating systems.

## Supplementary Material

Pais_et_al-ISAnalytics-Supplementary_Figures_bbac551

Pais_et_al-ISAnalytics-Supplementary_Table_1_bbac551

Pais_et_al-ISAnalytics-Supplementary_text_and_Methods_colors_bbac551

Pais_et_al-ISAnalytics-Extended_Data_1-tool_comparison_bbac551

Pais_et_al-ISAnalytics-Extended_Data_2-barcoding_bbac551

## Notes

**Daniela Cesana** is a biotechnologist, project leader in SR-Tiget in the unit of safety and insertional mutagenesis, with outstanding experience in vector safety and integration studies. She developed new technologies for integration site retrieval in liquid biopsies, for adeno-associated viral vector (AAV) characterization, and for off-target detection in gene editing applications.

**Maria Ester Bernardo** is a physician of the bone marrow transplantation unit at SR-Tiget, leading the gene therapy clinical program of MPSIH [ 8].

**Bernhard Gentner** is a physician-scientist and hematologist at SR-Tiget with long-lasting experience in hematopoietic stem cell manipulation and clinical development of gene therapy, principal investigator of the Hurler syndrome study (mucopolysaccharidosis type I, Hurler variant—MPSIH) [ 8].

**Eugenio Montini** is a biologist, head of the unit of Safety and insertional mutagenesis in SR-Tiget, with long-lasting experience in vector design and genotoxicity in gene therapy.

**Andrea Calabria** is in charge of molecular characterization and clonal tracking studies to assess the safety and efficacy of all institutional gene therapy clinical trials. His research focus is on deciphering the dynamics of the hematopoietic reconstitution in vivo after transplantation.
