# Supplementary material for: ISAnalytics enables longitudinal and high-throughput clonal tracking studies in hematopoietic stem cell gene therapy applications
Source: Brief Bioinform. 2022 Dec 21;24(1):bbac551. doi: 10.1093/bib/bbac551 (PMC9910212; doi:10.1093/bib/bbac551)

Supplementary Figure 1

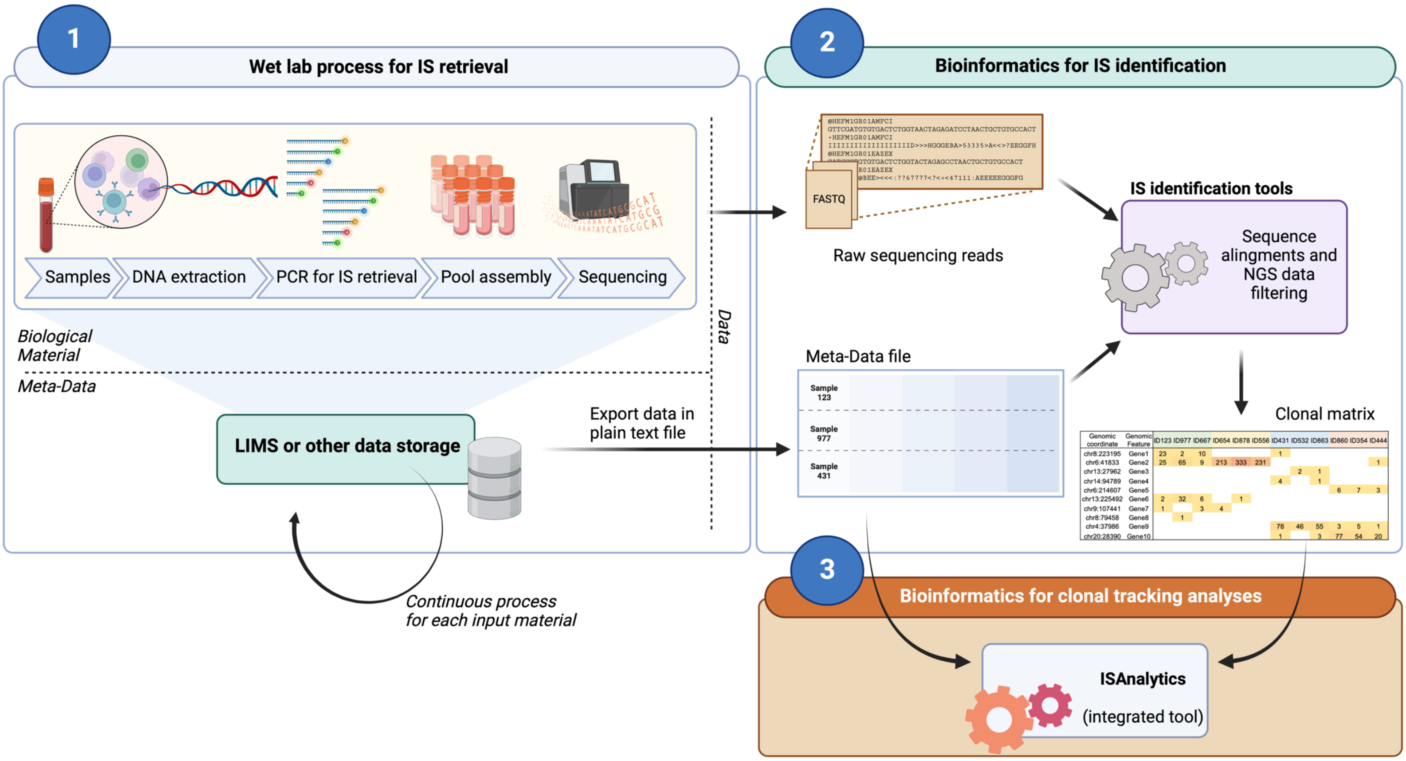

Supplementary Figure 2

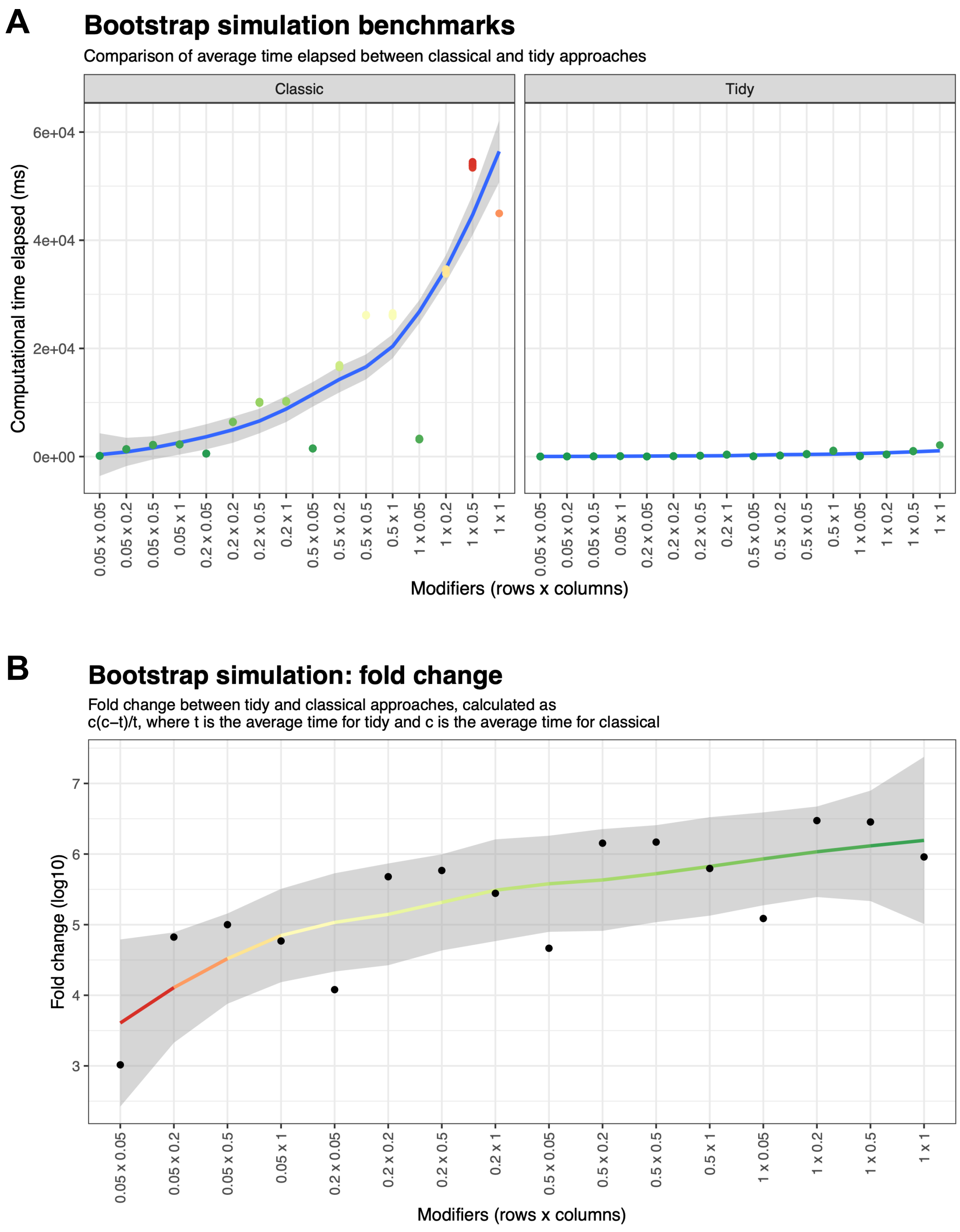

Supplementary Figure 3

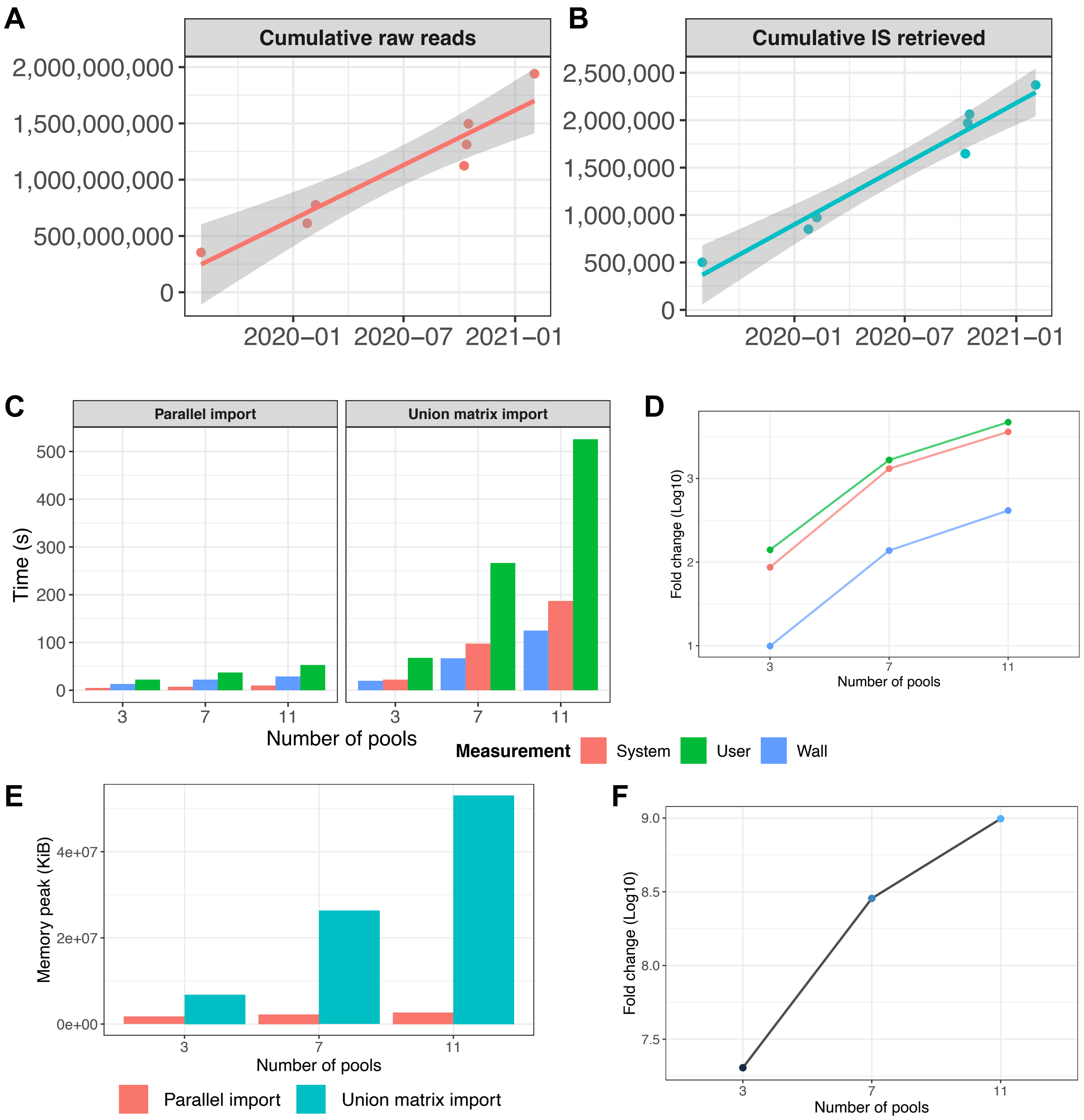

Supplementary Figure 4

A

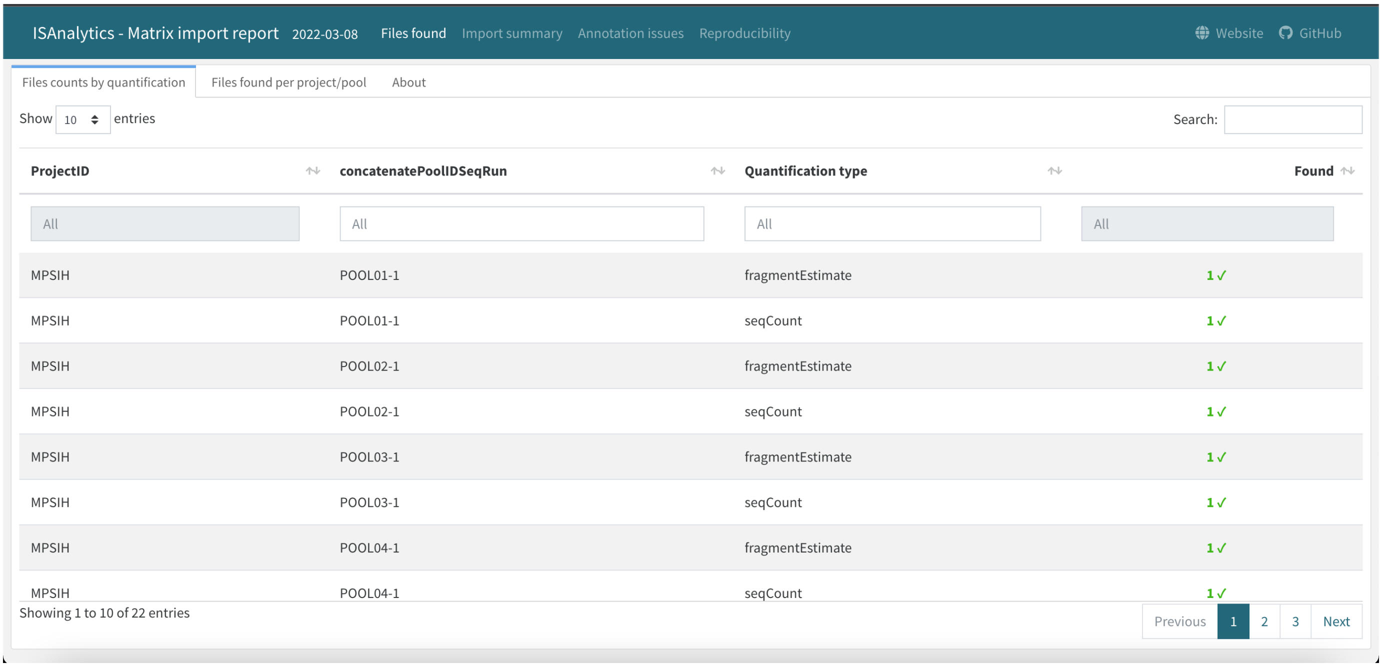

B

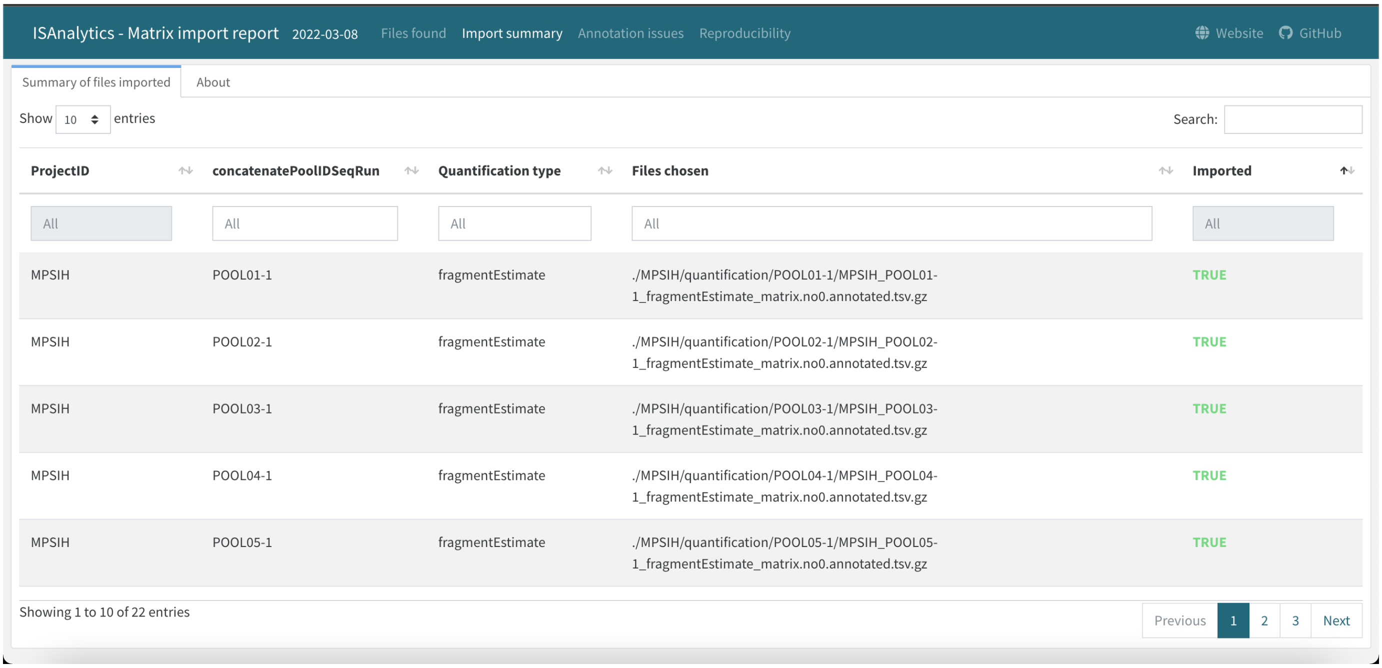

Supplementary Figure 5

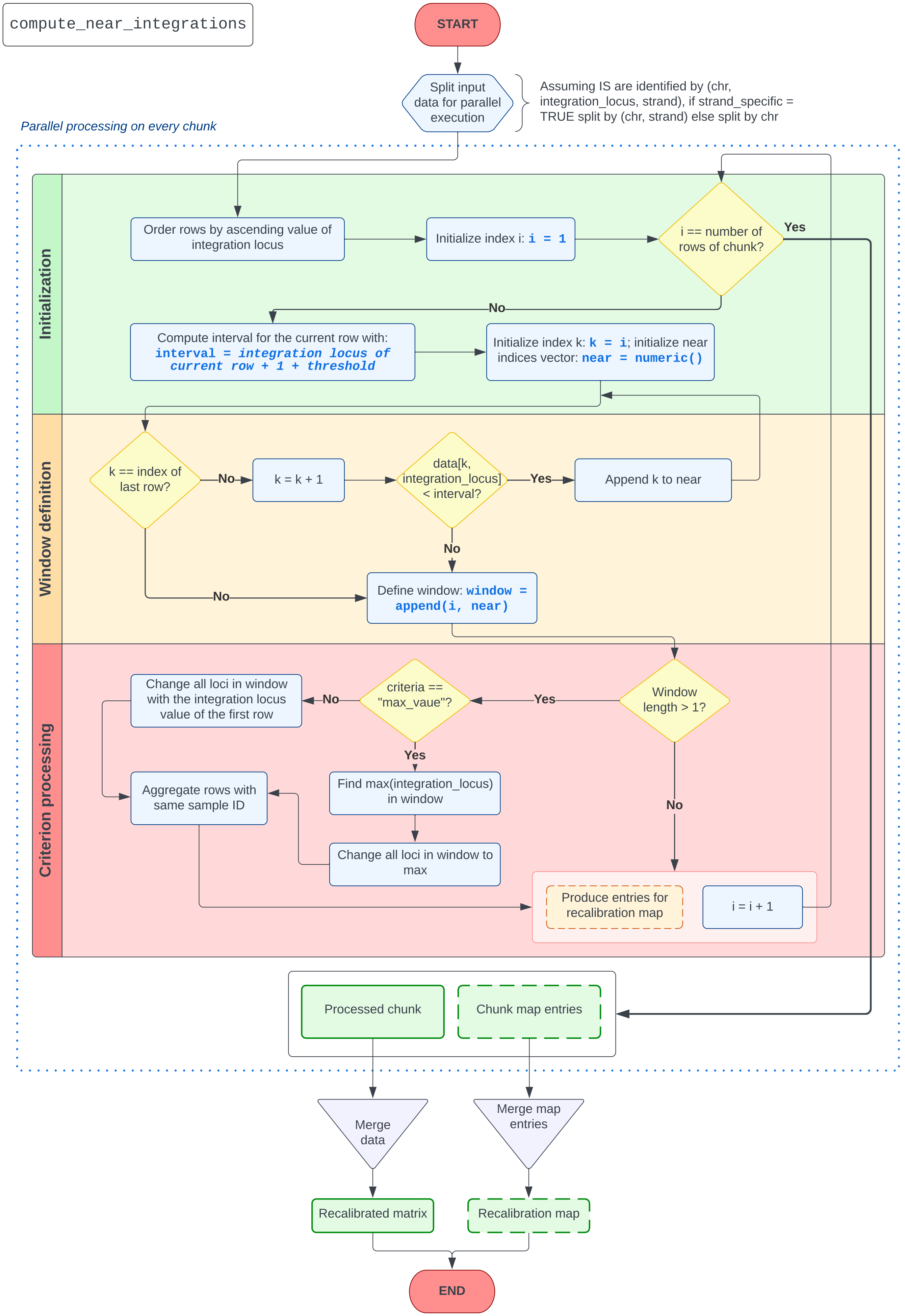

Supplementary Figure 6

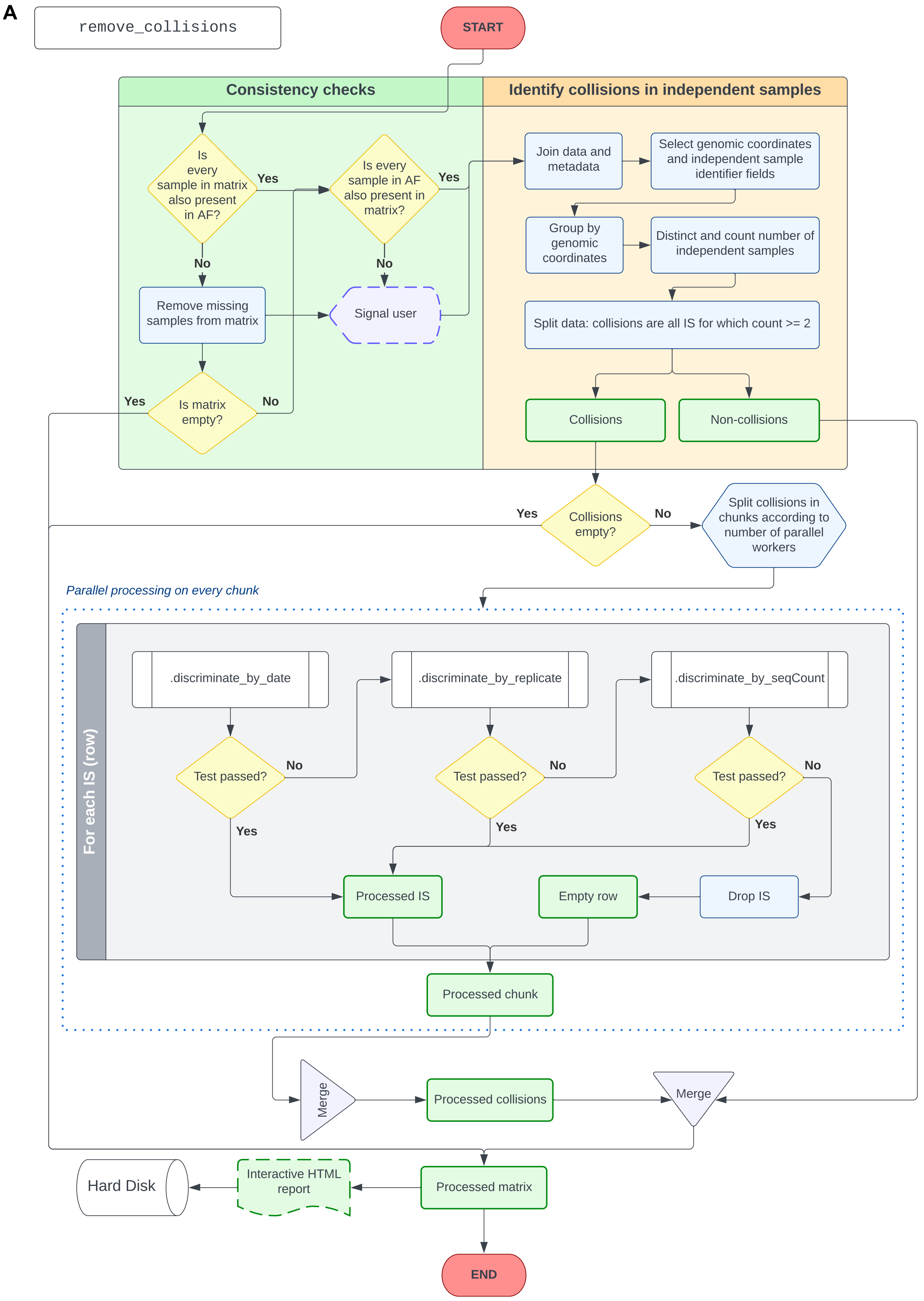

Supplementary Figure 6 (continued from the previous page)

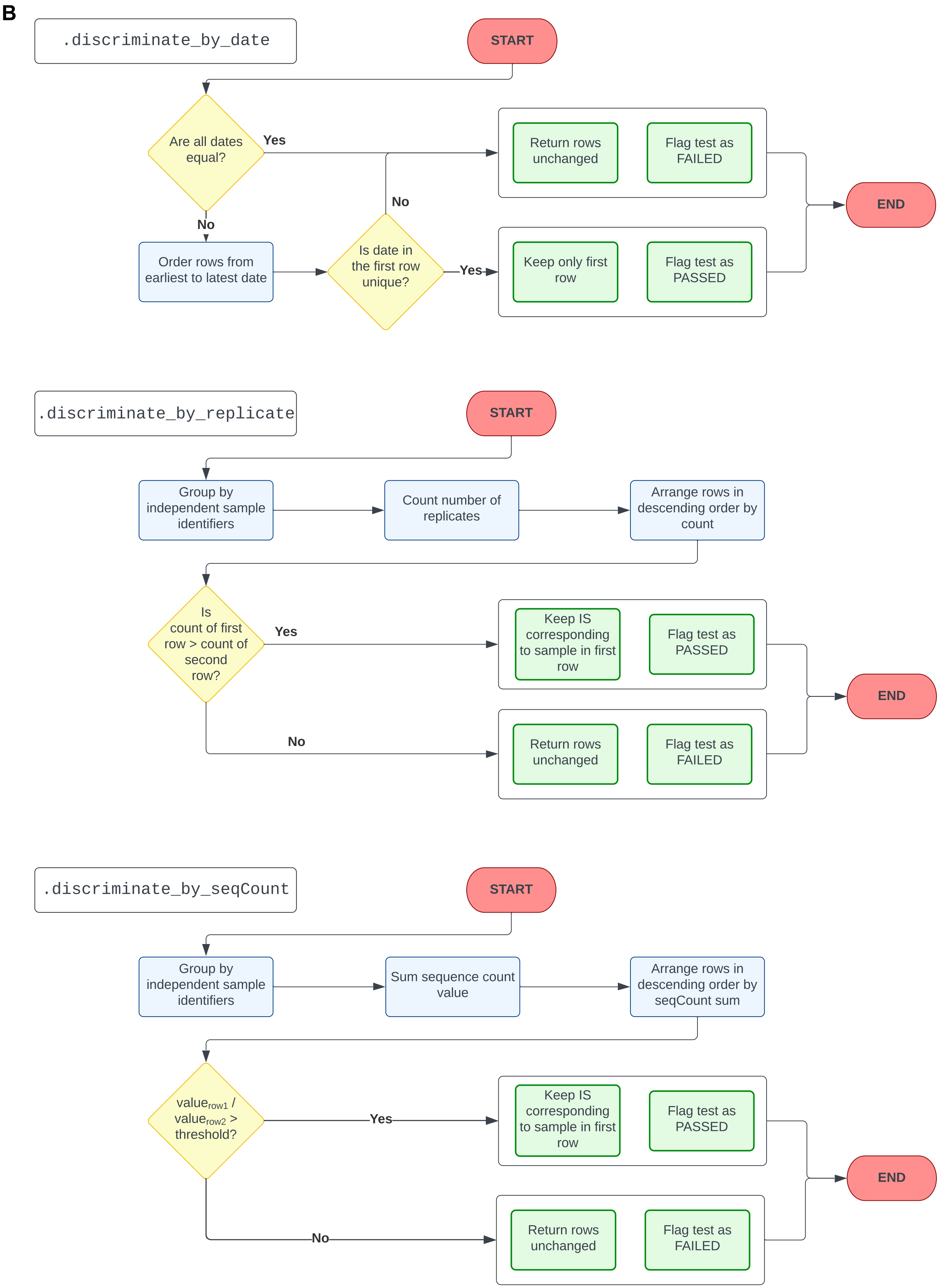

Supplementary Figure 7

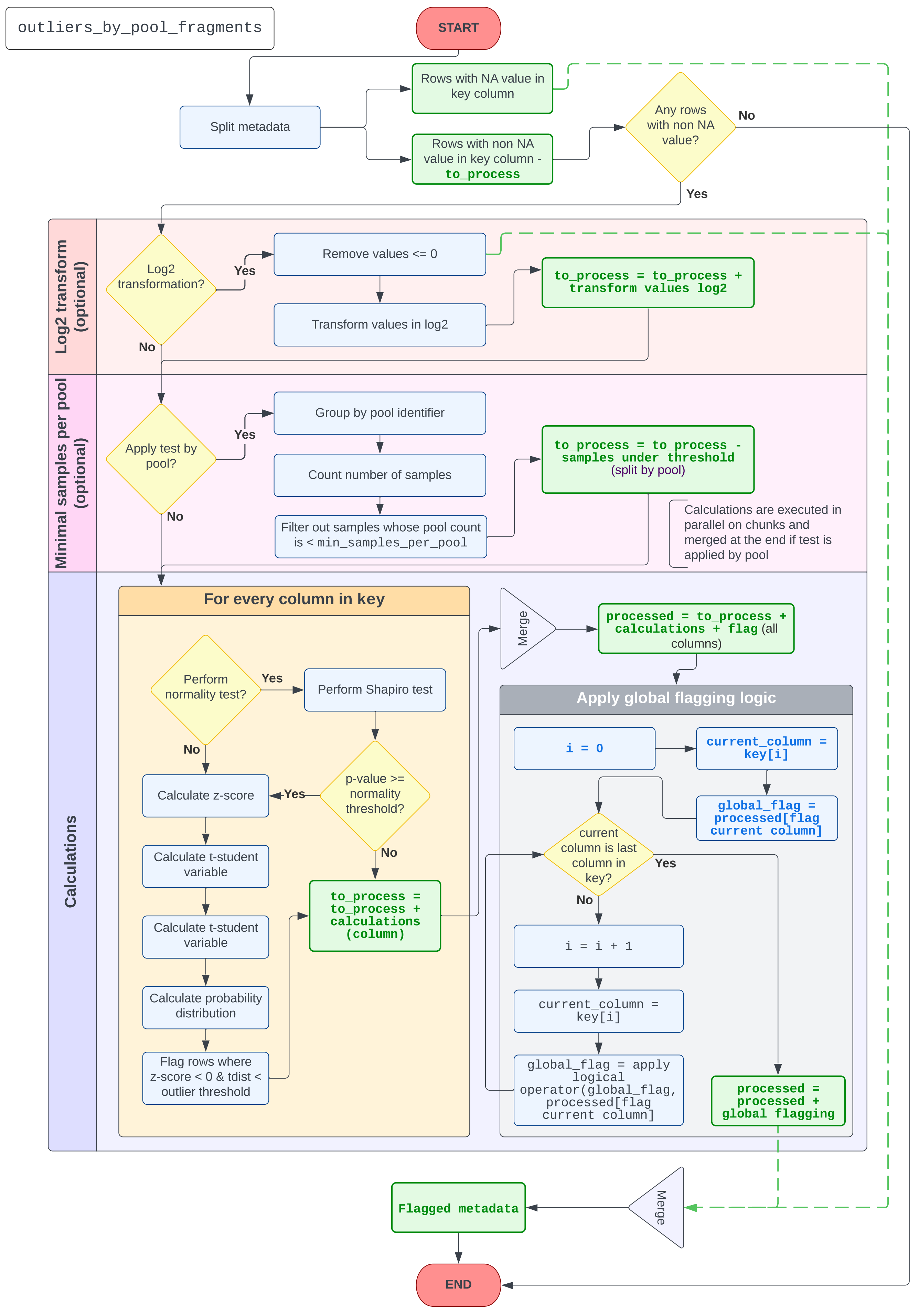

Supplementary Figure 8

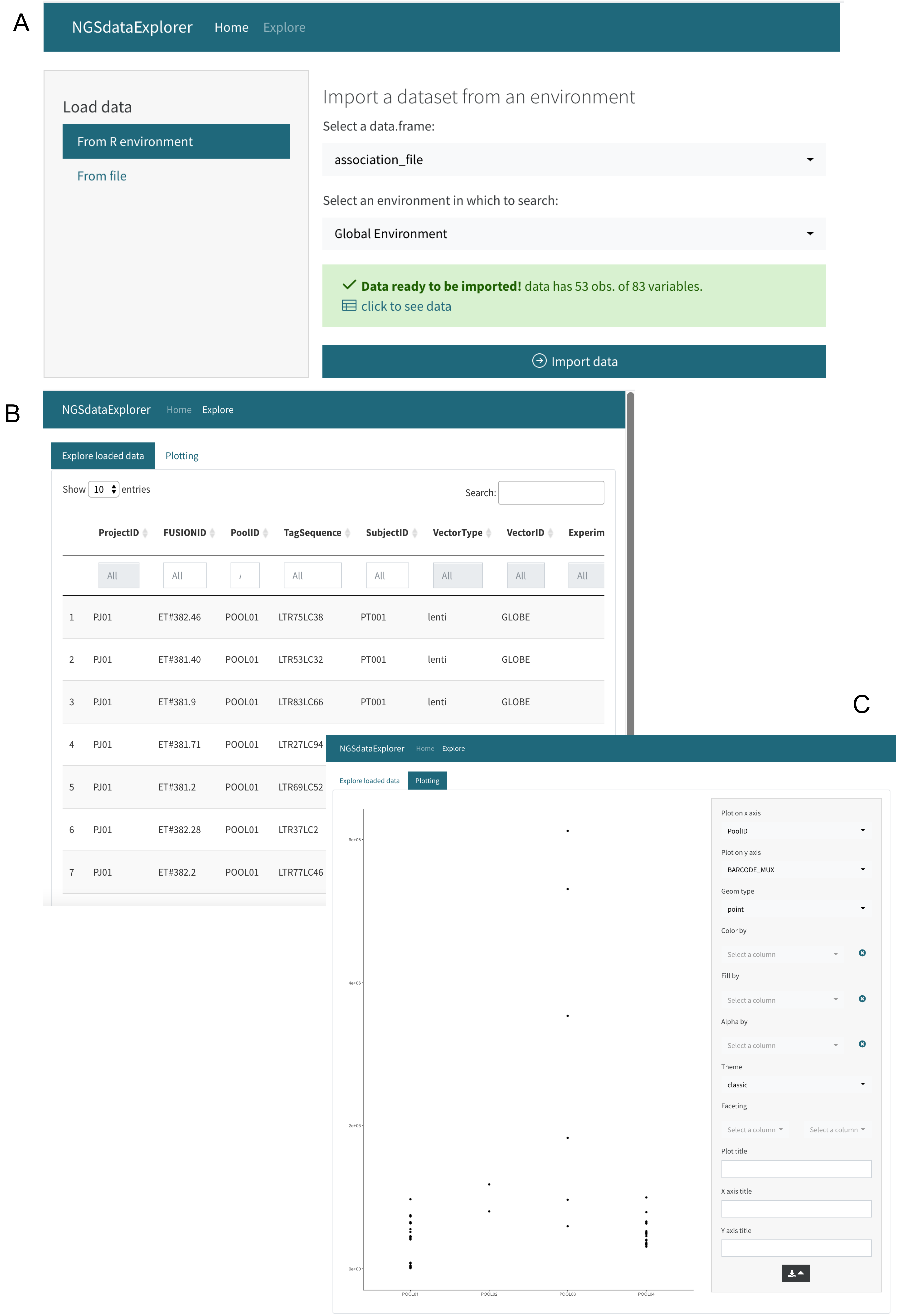

Supplementary Figure 9

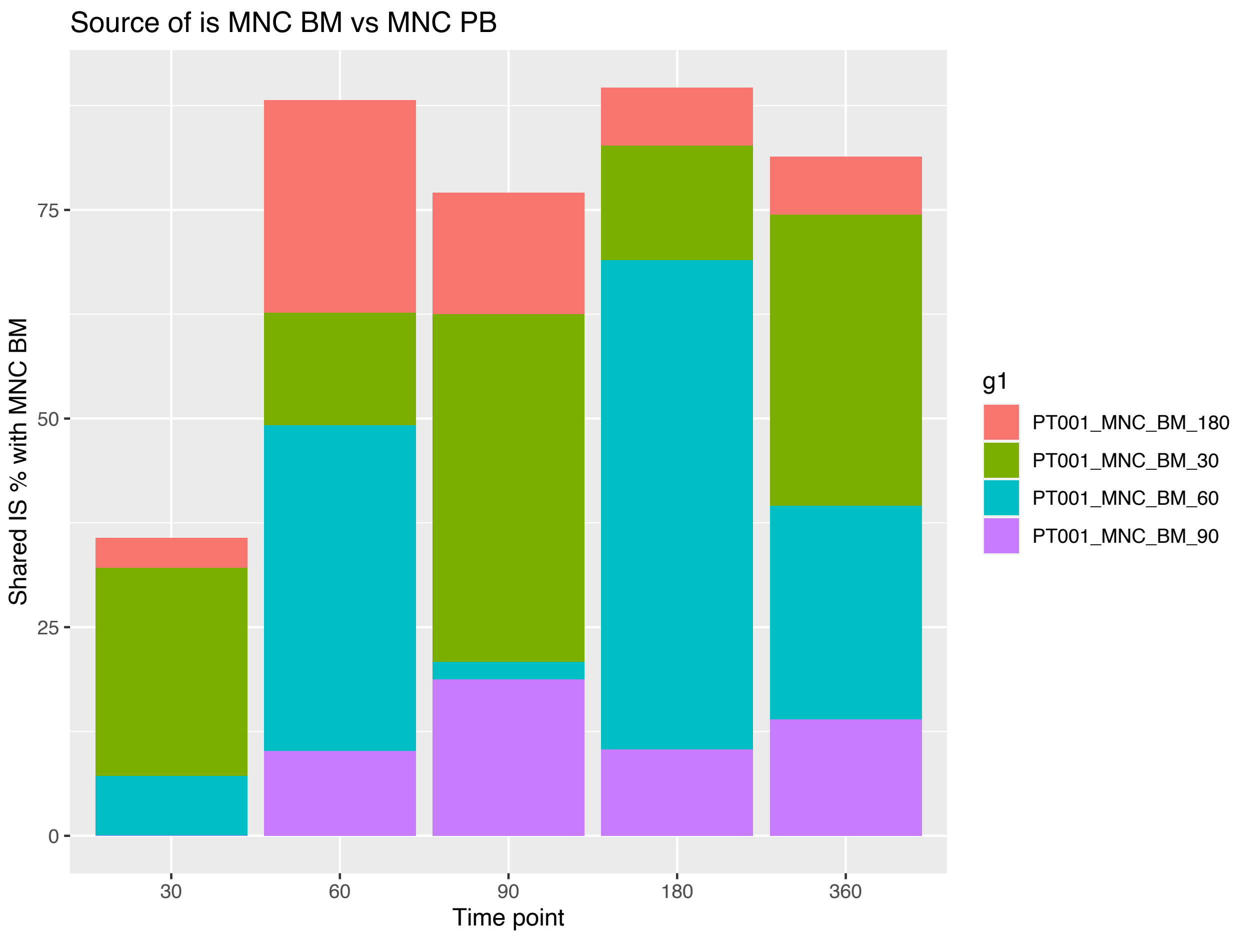

Supplementary Figure 10

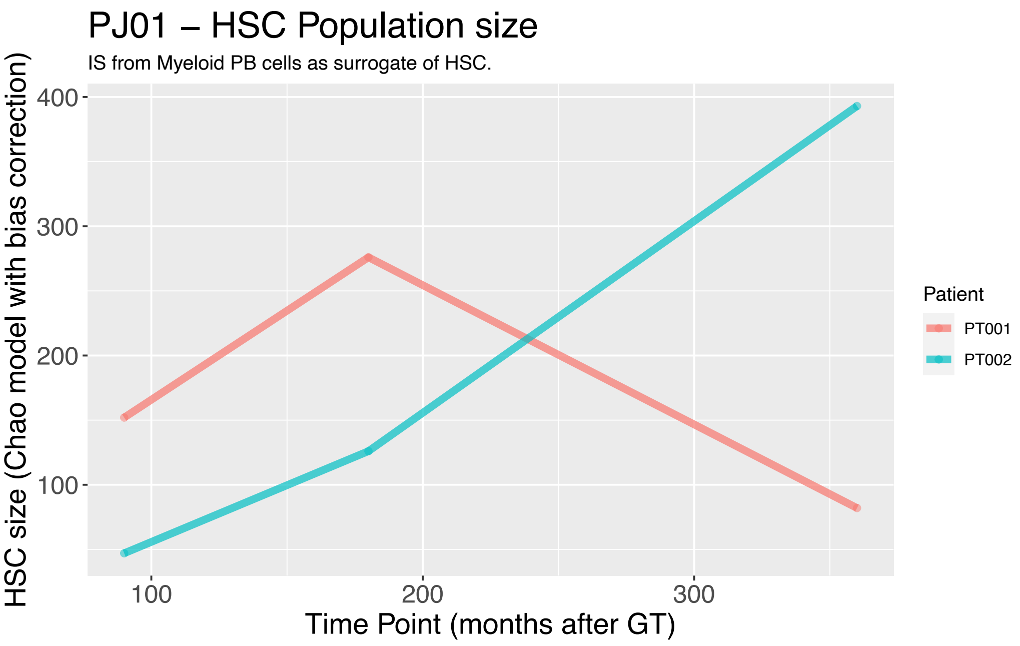

Supplement: Pais_et_al-ISAnalytics-Supplementary_Figures_bbac551 [file pais_et_al-isanalytics-supplementary_figures_bbac551.pdf]
