# Supplementary material for: ISAnalytics enables longitudinal and high-throughput clonal tracking studies in hematopoietic stem cell gene therapy applications
Source: Brief Bioinform. 2022 Dec 21;24(1):bbac551. doi: 10.1093/bib/bbac551 (PMC9910212; doi:10.1093/bib/bbac551)
Supplement: Pais_et_al-ISAnalytics-Supplementary_text_and_Methods_colors_bbac551 [file pais_et_al-isanalytics-supplementary_text_and_methods_colors_bbac551.docx]

ISAnalytics enables longitudinal and high-throughput clonal tracking studies in Hematopoietic Stem Cell Gene Therapy applications

Giulia Pais^1^, Giulio Spinozzi^1,#^, Daniela Cesana^1,#^, Fabrizio Benedicenti^1^, Alessandra Albertini^1^, Maria Ester Bernardo^1,2^, Bernhard Gentner^1^, Eugenio Montini^1^, Andrea Calabria^1,*^

^1^ IRCCS Ospedale San Raffaele, San Raffaele Telethon Institute for Gene Therapy (SR-Tiget), Milan, Italy; ^2^ IRCCS San Raffaele Scientific Institute, Vita-Salute San Raffaele University, Milan, Italy

^*^ Corresponding author (calabria.andrea@hsr.it) ; ^#^ equal contributing authors

Table of Contents

[Supplementary text 2](#_Toc118121011)

[Software release 2](#_Toc118121012)

[Supplementary Methods 3](#_Toc118121013)

[Data import 3](#_Toc118121014)

[Data structures and computational performances 6](#_Toc118121015)

[Preliminary benchmarks 7](#_Toc118121016)

[Recalibration 8](#_Toc118121017)

[Collision identification and removal 8](#_Toc118121018)

[Cumulative integrations 11](#_Toc118121019)

[Integration sites abundance 12](#_Toc118121020)

[Comparative analysis of clonal tracking tools 12](#_Toc118121021)

[Data pre-processing 13](#_Toc118121022)

[Descriptive statistics and diversity indices 13](#_Toc118121023)

[Clonal abundance 14](#_Toc118121024)

[Top *N* clones 14](#_Toc118121025)

[Clonal counts 15](#_Toc118121026)

[Exclusive features 15](#_Toc118121027)

[Clonal tracking with Genetic Barcodes 16](#_Toc118121028)

[Integrating ISAnalytics within a laboratory workflow 17](#_Toc118121029)

[Supplementary References 19](#_Toc118121030)

# Supplementary text

## Software release

ISAnalytics is an open-source R package, currently available both on the dedicated GitHub repository (<https://github.com/calabrialab/ISAnalytics>) and on Bioconductor (<http://www.bioconductor.org/packages/release/bioc/html/ISAnalytics.html>). ISAnalytics provides extensive and reproducible examples within in-depth documentation, as well as a dedicated and maintained website. Moreover, the package is developed following standard software development good practice, which include reuse of code whenever possible, usage of appropriate naming conventions, modularity and separation of concerns, and efficiency, which, in turn, make the software scalable, easily expandible and easier to maintain.

We also created a release, 1.8.0, and generated a docker to maximize reproducibility. The docker release can be downloaded from the following link in DockerHub: <https://hub.docker.com/r/giuliapais/isanalytics>. Moreover, we archived in Zenodo the ISAnalytics release 1.8.0 here described, both as software release (DOI: 10.5281/zenodo.7285314) and as docker (DOI: 10.5281/zenodo.7299338).

# Supplementary Methods

## Data import

ISAnalytics supports the analysis workflow from the very beginning and thus it includes dedicated and specialized functions for the import of different data sources.

ISAnalytics can import general sparse matrix (clonal tracking matrixes) as well as custom data matrixes structured with samples as columns and clones as rows.

Data import functions require specific file format, here reported. For the matrix of IS, the file format requires the following structure:

- Rows: list of ISs.
- Columns: the first columns (user defined) are dedicated to the genomic annotation of each IS, thus reporting the chromosome, the genomic position and orientation. The columns after the last annotation column refer to the observations (samples). Headers must be present, and each sample must have a distinct column ID from all the others. This column ID must also be present in the metadata file.

For the metadata file, samples can be described with user-defined columns. The only requirement for ISAnalytics is to have a concordant column of sample identifiers containing the same name reported in the column header of the IS matrix file.

We designed a full and detailed workflow in the package main page to support users on each import function and customization based on their own data: <https://bioconductor.org/packages/release/bioc/vignettes/ISAnalytics/inst/doc/workflow_start.html>

The file format of the IS matrix that ISAnalytics requires has been adopted in several studies. For example, recent barcoding studies for gene editing in HSC by Ferrari and collegues [3] released data matrixes of barcodes in the same input file format of the expected ISAnalytics files. Here we report the example of these import using the data uploaded in GEO with the following codes: GSE144340 (<https://www.ncbi.nlm.nih.gov/geo/query/acc.cgi?acc=GSE144340>), file name GSE144340_Matrix_542.tsv.gz. In this file, barcodes are listed in rows with their unique sequence as identifier (no genomic annotations are required) and the observations (samples) in columns with a unique ID name. A scratch of the data is here reported for simplicity:

| **BarcodeSeq** | **BM_A0** | **PB21_A0** | **BM_A1** | **PB21_A1** | **PB21_A2** | **PB21_A3** |
| --- | --- | --- | --- | --- | --- | --- |
| AAAAAAAATTTTTAAACGTACC | 1 | 0 | 0 | 0 | 0 | 0 |
| AAAAAACATATCTATAGTTACC | 1 | 0 | 0 | 0 | 0 | 0 |
| AAAAAAGACGACGATAGGCACG | 0 | 0 | 0 | 0 | 0 | 0 |
| AAAAAAGACGTTTATAGGTGTA | 0 | 0 | 0 | 0 | 1 | 0 |

Since no metadata file is directly associated with this file, we can design a custom file only for testing purpose as follows, containing the ID column with names corresponding to the matrix data file:

| **ProjectID** | **SubjectID** | **Tissue** | **TimePoint** | **CellMarker** | **ID** |
| --- | --- | --- | --- | --- | --- |
| PMID32601433 | A0 | BM | 21 | Whole | BM_A0 |
| PMID32601433 | A0 | PB | 21 | Whole | PB21_A0 |
| PMID32601433 | A1 | BM | 21 | Whole | BM_A1 |
| PMID32601433 | A1 | PB | 21 | Whole | PB21_A1 |
| PMID32601433 | A2 | PB | 21 | Whole | PB21_A2 |
| PMID32601433 | A3 | PB | 21 | Whole | PB21_A3 |
| PMID32601433 | A4 | BM | 21 | Whole | BM_A4 |
| PMID32601433 | A4 | PB | 21 | Whole | PB21_A4 |
| PMID32601433 | C0 | PB | 21 | Whole | PB21_C0 |
| PMID32601433 | C1 | BM | 21 | Whole | BM_C1 |
| PMID32601433 | C1 | PB | 21 | Whole | PB21_C1 |
| PMID32601433 | C2 | BM | 21 | Whole | BM_C2 |
| PMID32601433 | C2 | PB | 21 | Whole | PB21_C2 |
| PMID32601433 | C3 | BM | 21 | Whole | BM_C3 |
| PMID32601433 | C3 | PB | 21 | Whole | PB21_C3 |

Once imported the two files, custom flows of data analysis can be developed, for example performing column statistics (including entropy based measures or clonal distributions), data aggregation by mouse ID (column SubjectID) with updated statistics and clonal abundance. The full example with the corresponding code is reported as Vignette in Bioconductor.

Coming back to gene therapy applications producing IS datasets with similar file format, we propose one of the recent HSC clinical trial based on gamma retroviral vector for Wiskott-Aldrich syndrome by Wunsche and colleagues presenting long-term follow-up of transplanted clones [2]. Along with the publication of the results, the authors released the matrix file (Patient_IS_detailed.xlsb) containing all IS. The file format is in line with the required input files accepted by ISAnalytics.

These reasons led us to envisage ISAnalytics to be broadly useful in many different applications dealing with clonal tracking technologies. Further extensions for other input files can be implemented.

Moreover, ISAnalytics is able to detect the presence of a standard folder structure produced by VISPA2 when providing an appropriate root folder and project folder column. At the end of the import procedure, an automatic report is generated to summarize the list of acquired files, the summary statistics of clonal data and samples, and any potential error/exception raised during this step. The report is saved as an interactive web page (HTML self-contained page) with a summary dashboard.

Automated IS matrix import is based on a *divide et impera* approach such that, given a user-defined number of parallel jobs/workers to use, each file listed in the metadata file is processed by a specific job (asynchronously) which imports the corresponding data matrix and converts the matrix into a tidy data structure via a pivoting operation. At the end of all jobs, all data is merged in a single tidy structure and a new interactive report is produced (if requested by the user) containing the details of all pool matrices imported and list any issues/errors. The same method is applied for the import of pool statistics files, which are produced automatically by VISPA2 for each sequencing pool and contain information on the quality and number of reads.

Data structures and computational performances

Sparse data are characterized by a high proportion (98-99 % on average) of non-significant elements and, if acquired as data frames, provide many computational issues in memory load and computational cost of data processing, reflecting in the scalability of clonal tracking analyses. We decided to exploit an efficient data structure that we identified in the R tidy[1] in which non-significant values are absent with a lower memory footprint, optimized for relational algebra operations and database-like operations. Moreover, we designed a fully parallel import with a *divide et impera* approach in which ISAnalytics loads and converts each sparse matrix as tidy structure and asynchronously merges the data into a unique data structure.

To benchmark the performance of the tidy data structure and quantify the efficiency compared to data frames, as used in other available tools, we used simulated data generated to replicate real case scenarios and randomized with bootstrap to obtain results of different data size. We run an overall number of 151 simulations given input sparse matrixes with a variable range of 16965 - 339301 rows (corresponding to clones) and 20 - 293 columns (corresponding to samples) (**Supplementary Table 1**, and **Methods** for details). For each input matrix, we run the import procedures and the column data aggregation for 30 rounds of benchmarks to test the computational time. Our results (**Supplementary Figure 2A**) showed a clear improvement in the parallel tidy structure with respect to the standard data frames (used with “apply” operations in the aggregation procedure) since across all simulations we observed a linear but minimal increase in computational time for the tidy results whereas sparse matrix with data frames exponentially increases along with the incremental bootstrap simulations. We compared the two approaches in terms of fold change (tidy on sparse data frame) to quantify the speed-up and our results showed a major increase ranging from 3 to 6.5 logs (**Supplementary Figure 2B**) (10^3^ for smaller datasets whereas 10^6^ for larger datasets, with an average fold change lying between 10^4^ and 10^5^).

## Preliminary benchmarks

Preliminary benchmarks were conducted to test the performance of the tidy approach for frequently used operations such as the aggregation. We used an input dataset that replicated a clonal tracking dataset of a medium size, composed of 288 columns (samples) and 339301 rows (clones) and with 5 extra columns containing genomic coordinates and annotations for IS.

Given the initial dataset, we decided to test the performances by random subsampling rows and columns with a bootstrap approach, from 100% to 5% (100%, 50%, 20%, 5%). For each subsampling, 10 randomizations were drawn with the exception of 100% x 100%, corresponding to the original matrix (**Supplementary Table 1**) for a total of 151 samples. Annotation columns are always included and kept as they are. We then run the aggregation function for each input dataset using the standard data frame import of the sparse matrix and the use of tidy data structure. Performance in terms of computational time was monitored through the R package “microbenchmark”. The full code is available in the supplementary materials. All benchmarks were run on a workstation with the following specifications: Intel(R) Xeon(R) CPU E5-2690 0 @2.90GHz with 32 cores, 250 GB of RAM, OS Ubuntu 20.04.3. The version of R and packages used is listed below:

- R version 4.1.0
- *dplyr* version 1.0.6
- *magrittr* version 2.0.1
- *rlang* version 0.4.11
- *tidyr* version 1.1.3
- *tibble* version 3.1.2
- *microbenchmark* version 1.4-7

## Recalibration

Once loaded files in ISAnalytics, IS recalibration is required when different sequencing libraries are imported and merged. From each library, each IS is placed on a specific locus depending on the alignment position of the supporting reads. Since reads may present some artifacts even at the putative locus of the IS, alignments may reflect those artifacts and the corresponding IS could start with a few base pair differences (usually in the range of 3 bases, as previously reported [4]). Thus, single ISs sampled in two independent sequencing libraries may not present the same locus although referring to the same IS. If we combined the two lists of IS only by genomic positions without correcting this misplacement, we could end up with two independent IS, introducing false positive IS. To prevent this issue, we developed a recalibration function, called “compute_near_integrations”, that adjusts the genomic coordinates (placing the IS at the base with the highest number of mapped reads) (see the algorithm in **Supplementary** **Figure 5**).

## Collision identification and removal

With the term “collision”, GT studies refer to identical IS retrieved in two independent samples; this case has a very low probability and for this reason one of the two samples is considered contaminated by the other sample often caused by PCR artifacts as reported in previous studies[5, 6]. In ISAnalytics we developed the function for collision removal called “remove_collisions” (**Supplementary** **Figure 6** for algorithmic details). Briefly, samples are analyzed by their date of processing (reported in the metadata file) and IS are assigned to the patient that tracked the IS at a higher frequency and at a higher number of reads. The function summarizes the results in an interactive report (**Figure 3B**).

More in details, in the metadata file, independent samples are defined by a combination of several fields (super-key), such as k-tuple:

$$independent sample=({field}_{1}, {field}_{2},\ldots, {field}_{k})$$

In the vast majority of the cases, to identify independent samples is enough using 2 fields: the project identifier and the subject identifier.

$$independent sample=(Project ID, Subject ID)$$

Given $n$ independent samples, ${(Project ID, Subject ID)}_{1},\ldots,{(Project ID, Subject ID)}_{n}$

$$x is collision \Longleftrightarrow x belongs to 2 to n independent samples$$

After a first step of identification, collisions events are either reassigned or removed, depending on the outcome of a 4-step-test:

1. The first test verifies if it is possible to identify a unique maximum date (by default sequencing date): with unique maximum we mean the date must be earlier than any other date for that collision event and must not be duplicated. If the outcome is positive, then only the integration event associated with that date is kept, all others are considered contaminations and are discarded. If there is no maximum the outcome of the test is negative, and the algorithms proceeds with check 2.
2. Since for each sample we usually have at least three PCR replicates, the second test checks, for each independent sample, in how many of these replicates the event occurs. If it is possible to single out a maximum replicate count between all the independent samples involved, all integration events that do not match are dropped. However, if there is no count dominating among others, the test fails, and a further step is required.
3. In the third test the value of the sequence count quantification is considered: for each independent sample, a cumulative value of the quantification is computed by summing individual values. The values are then sorted in descending order and the biggest value is compared to the second biggest: if the ratio between the 2 is above a certain threshold (by default 10), the event associated with the greatest sequence count is kept and all others discarded. If, however, the value does not exceed the threshold, no decision can be made, and the algorithm moves to the final step.
4. Finally, if none of the tests were successful, all integration events associated with the collision event are considered contaminations and are dropped from the final matrix.

Identification and removal of sample outliers

Within each sequencing library, samples may have a different number of reads (raw data reads), and users would like to remove the samples that returned a number of raw reads significantly lower than expected. To address this point, we designed a filtering function (“outlier_filter”) and an associated outlier test function (“outliers_by_pool_fragments”) (details in **Methods** and **Supplementary Figure 7**) that analyzes the number of raw reads reported by each sequencing library, identifies the expected number of reads per sample (as the average number of raw reads in the pool) and performs a Z-score statistics to report the variance of each sample on the mean. Samples resulting in a variance below a user-defined threshold (default at 3) are labeled and removed. At the end of the procedure, ISAnalytics returns interactive reports that allow a fine revision and check of all the steps (**Figure 5C**).

The sample pseudo-code for the outlier removal function is here reported:

for each pool in pool_list:

if pool has < 5 replicates:

go to next pool

else:

transform BARCODE_MUX col in log2

calculate zscore of log2(BARCODE_MUX) column

calculate values for the random variable t using

the t-student distribution formula

calculate the density distribution

flag samples if tdist < threshold AND zscore < 0

Indeed, the function allows more flexibility in the choice of the column(s) to consider for calculations, and additional options: a normality test can be performed before any other calculation with the Shapiro-Wilks test, log2 transformation of points can be skipped, the threshold can be adjusted according to user needs and if the test has to be done on more than one column, there is the possibility to specify the logical operator(s) to apply for the final flagging.

Shiny interface for NGS data exploration

Among the functionalities included, the possibility of importing data is featured, either directly from R environments or from tabular files, and interactive data filtering and searching. In addition, a dedicated section of the application allows flexible and versatile plotting options with customizable parameters for selecting x- and y-axis variables, and aesthetics mappings and faceting. Finally, it is possible to export the plotted data in various file formats (.png, .pdf, etc.). The application can be easily run by calling the function “NGSdataExplorer()”.

Cumulative integrations

The function cumulative_is accounts for the number of new clones observed at each new sampling (usually time points). The cumulative function is useful to study if and when the number of observed clones is representative of the patient under analysis (if dealing with clinical trials): if the number of new clones is significantly decreasing over time it means that the observed repertoire of clones is enough to describe the patient and thus to characterize the hematopoietic reconstitution *in vivo*. The cumulative_is function implements this logic by collecting for each time point the number of clones observed at that time point and adding (in union) the clones observed in all previous time points. The function returns the incremental counts for each group in input and/or the actual genomic coordinates for the integration events. The output is obtained by splitting the input data by time point, ordering chunks from the earliest to latest one, and performing an incremental set union through a reduce operation (internally executed via the function “accumulate” of the package “purrr”). The value of the quantifications is dropped since no inference on values is performed.

## Integration sites abundance

Quantification of the abundance of each clone is performed by calculating the relative percentage of cells (if dealing with number of genomes with the fragment estimate) or reads (if dealing with sequencing reads) of each clone over the total number of cells/reads for a specific sample.

Given an integration event $x_{k}$ with an associated quantification value in group $y$, where $y$ is defined by an arbitrary number of metadata fields and contains $n$ integration events, the abundance value for $x_{k}$ is calculated as:

$$a_{{jx}_{k}}=\frac{{value}_{x_{k}}}{\sum_{i=1}^{n} {value}_{x_{i}}}$$

Once obtained the data matrix of clonal abundances, values can be plotted using a streamgraph representation, implemented as a series of alluvial plots to track the clonal expansion overtime or over tissues using the function integration_alluvial_plot. Alluvial plots are internally realized with the aid of the package “ggalluvial”. The function also has the option to produce a color-coded table with the top 10 most abundant genes internally generated using the package “gridExtra”.

## Comparative analysis of clonal tracking tools

To compare the set of functionalities offered by both ISAnalytics and barcodetrackR, we deemed appropriate to write a simple R script to show how common operations can be carried out with the two approaches. The full R script is available in the **Extended Data 1**.

We used the data released in ISAnalytics R package as inputs to both packages’ functions.

### Data pre-processing

As already mentioned in **Table 1**, ISAnalytics includes a set of functions dedicated to data cleaning and pre-processing that are not available in barcodetrackR: in particular, in the R script, we showed the recalibration step, the removal of outliers from metadata, the removal of collisions and the aggregation of data by metadata fields.

### Descriptive statistics and diversity indices

Both tools allow for calculating sample statistics and obtaining plots of clonal diversity; however, data inputs and methodologies slightly differ. While the ISAnalytics function accepts data in the tidy format and calculates at the same time an arbitrary set of statistics (including Shannon diversity index, Simpson and inverted Simpson indexes at the same time), the “clonal_diversity” function in barcodetrackR expects data input in sparse file format only, more precisely in a Summarised Experiment structure, and can return a data frame containing only one of the specified indices at time. The following steps were needed to convert our data structures into compatible inputs for the function:

1. From the aggregated tidy matrix, obtain a sparse matrix.
2. Unite all the genomic coordinate columns in a single column “id”.
3. Fill NA values in the columns with zeros.
4. Convert the column “id” into row names.
5. From aggregated metadata, unite all columns that formed the aggregation sample key in the column “SAMPLENAME” (as documented in the barcodetrackR package).
6. Obtain a compatible dataset with the function “create_SE”.

To obtain the statistics results in tabular format with barcodetrackR it is required to launch the function “clonal_diversity” with the argument “return_table=TRUE”; setting the argument to FALSE would yield a plot object. With data produced by ISAnalytics, the plot can be obtained by simply calling the appropriate ggplot2 functions on the produced statistics in tabular format.

### Clonal abundance

Both packages provide functions to calculate and plot clonal abundance, but they present some differences: besides the input format already mentioned in the previous paragraph, data in tabular format can be obtained in ISAnalytics with the function “compute_abundance” and in barcodetrackR with “rank_abundance_plot” by setting the argument “return_table=TRUE”. While the percentage values are exactly the same, barcodetrackR adds 3 more columns of data: cumulative abundance, rank, and scaled rank, which are not present by default in ISAnalytics output. However it is worth mentioning that the very same structure can be obtained with a simple dplyr::mutate operation on ISAnalytics output.

The approaches for plotting the data however differ: while barcodetrackR plots the cumulative value of the abundance for each sample by rank, ISAnalytics leverages on abundance data to obtain the so-called alluvial plots or streamgraphs. In addition, barcodetrackR provides one more function, “clonal_contribution” which can be used to plot abundance data in the form of area plots or stacked bar plot, however we were not able to make it work with our data.

### Top *N* clones

While both ISAnalytics and barcodetrackR include functions to compute and identify the top N clones, the approaches and the quantities, as well as plotting methods differ.

ISAnalytics provides the function “top_integrations”, which is flexible in terms of parameters and inputs: generally, we are interested in tracking the top N abundant clones, so we provide in input the abundance table and we specify the name of the column containing the values to rank, however this function works with any other kind of data that contains genomic coordinates and the columns of the sample identifier. Moreover, the function “top_integrations” can calculate both the top N clones overall or for each sample. The output is in tabular format and can easily be plotted with ggplot2 functions according to user needs. Additionally, when working with abundance and alluvial plots, by specifying the argument “top_abundant_tbl = TRUE” when calling the function “integration_alluvial_plot”, we can obtain tables with colored cells containing the top N abundant genes, where colors are linked to the colors of the streams (ribbons and stacked bars) in the alluvial plot. In barcodetrackR, the approach is different since calculations are done in terms of distance measures and are visualized with hierarchical heatmaps via the function “barcode_ggheatmap”. Although the documentation states that the top N clones can be tracked over time, there is no direct argument to specify where the time information should be taken/acquired, therefore leaving to the user the duty to appropriately choose the samples when tracing the heatmap.

### Clonal counts

Simple clonal counts (the number of distinct IS) and cumulative clonal counts over time can be calculated and plotted by both tools. With ISAnalytics for clonal counts, we simply called ggplot2 functions on descriptive statistics obtained before, whether barcodetrackR explicitly provides the same functionality with the function “clonal_count”. Cumulative IS can be obtained in tabular format in ISAnalytics via “cumulative_is” and then plotted as before, while in barcodetrackR this is possible by setting the argument “cumulative = TRUE” in the previous function. However, it is worth noticing that the results of cumulative numbers of IS differ with the 2 approaches, most likely due to limitations in the number of fields on which calculations are performed.

### Exclusive features

Despite the common functionalities, the two tools include a set of different operations and functions that are exclusive to each other. barcodetrackR provides a set of functions for sample correlation and distance measures, and for sample bias analysis in addition to a dedicated function for chord plots. Although the vast majority of the functions correctly returned results, we experienced several errors in other functions which unfortunately we were not able to fix nor determine the specific causes.

On the other hand, ISAnalytics provides functions dedicated to lineage bias analysis, based on the sharing of integration sites between different samples and that can be plotted in different ways, common insertion sites (CIS) statistics both overall and over time, population size estimates with capture-recapture models, identification and visualization of waves of clones over time and a circos plot visualization of genomic density (as described in other sections).

## Clonal tracking with Genetic Barcodes

In this section we will present how to use ISAnalytics in another application context such as barcoding studies. The dataset is the same as reported in the “Data Import” section (Ferrari S. et al, Nature Biotechnology 2021) [3] that exploited the files with Gene Expression Omnibus (GEO) accession code GSE144340 and file name GSE144340_Matrix_542.tsv.gz. All results are then reported in the R code and markdown in **Extended Data 2** and within the online Bioconductor Vignette “Getting started with ISAnalytics”. The following file adjustments are required to import the original matrix file: (1) headers were shifted 1 column to the right; (2) the first column header was named “BarcodeSeq”.

The aim of this test was to import a different data type and perform the following downstream analyses: (1) to process descriptive statistics with clonal distribution and population entropy under all our indexes; (2) to perform data aggregation by mouse ID both for the clonal matrix and the metadata file; (3) to process descriptive statistics on the aggregated dataset, including population diversity; (4) to compute clonal abundance on the aggregated dataset.

After setting the appropriate dynamic variable configurations, as explained in the package vignette “Setting up the workflow and first steps”, we proceeded with the import of both metadata and data, obtaining the classical tidy data structure and descriptive statistics for each sample identifier. It was subsequently possible to perform an aggregation step and to obtain both aggregated descriptive statistics and clonal abundance. Of course, specific operations that require the presence of genomic coordinates in data (such as common insertion sites and collision removal) are not applicable in this use case, hence we did not show them. Since our aim was to show the applicability of ISAnalytics to another domain and how potentially exploit the available functionalities rather than to present a new case study in another clonal tracking biological platform, we did not produce any further biological analyses nor plots, leaving to interested users testing their own hypotheses and biological questions.

## Integrating ISAnalytics within a laboratory workflow

Here we describe how to integrate ISAnalytics in a laboratory workflow for IS retrieval and analysis (**Supplementary Figure 1**). In a laboratory dealing with IS, each input sample is processed to isolate DNA and perform PCR procedures to isolate and amplify the genomic portions containing the vector-host genome junctions. If the laboratory is supported by automation and equipped with management software, all steps of the experimental procedures that elaborate the biological material are usually recorded by a dedicated LIMS, or alternatively registered in other databases such as spreadsheet files, tables, or even flat files. At the end of the experimental procedure, all samples are processed and then assembled to be sequenced, obtaining a sequencing file of raw reads (FASTQ file format). The metadata recorded include sample details (patient ID, tissue, cell marker, etc.) and the experimental details of the sample processing (from sample ID, to the amount of DNA used for each PCR reaction and the residual material, the sequencing date, etc.). To maximize the flexibility of system integration, ISAnalytics is not restricted to any specific LIMS solutions nor forcing/limiting to adopt LIMS and automation, rather ISAnalytics takes as input a flat file of metadata that can be generated by any data sources. Nevertheless, having a LIMS that stores and manages all metadata well organized and maintains data reproducibility would allow an easier connection with ISAnalytics by exporting the flat file of metadata to ISAnalytics. The same bridge would be required while running the second step of the workflow which is the bioinformatics IS identification from the FASTQ raw sequencing files and the metadata files here used to set up the proper barcodes for the sample demultiplexing. IS identification can be realized by several available tools, from VISPA2 to INSPIIRED, as already mentioned. ISAnalytics is fully integrated with VISPA2, meaning that all the output files from VISPA2 and its file system are recognized by ISAnalytics and automatically acquired. This is a great advantage while setting up a fully automated laboratory workflow since the outputs of VISPA2 are not exclusively limited to the clonal matrix but rather describe all the intermediate steps of analysis, from the trimming procedure to the alignment and filtering, up to the final list of the reads of IS with the clonal matrix in several quantifications methods (by number of sequencing reads, number of different lengths of the mapped fragments, number of cells estimated by SonicLength, and number of unique molecular identifiers). Indeed, ISAnalytics can explore, load, and analyze all VISPA2 files and generate automated web reports before running any downstream analyses for clonal tracking. Of course, if a laboratory is using its own tool for IS identification, the only requirement is related to the output clonal matrix that must be formatted as described in the Data import section. The final step of bioinformatics data analysis is directly involving ISAnalytics to process clonal matrix together with the metadata file to address specific biological questions defined by the users. It is worth noticing that the same procedure can be adapted if the laboratory is performing barcode tracking studies, for which we report in the data import section some example of usage of ISAnalytics.

# Supplementary References

1. Wickham H. Tidy Data, Journal of Statistical Software 2014;59:1 - 23.

2. Wünsche P, Eckert ESP, Holland-Letz T et al. Mapping Active Gene-Regulatory Regions in Human Repopulating Long-Term HSCs, Cell Stem Cell 2018;23:132-146.e139.

3. Ferrari S, Jacob A, Beretta S et al. Efficient gene editing of human long-term hematopoietic stem cells validated by clonal tracking, Nat Biotechnol 2020;38:1298-1308.

4. Spinozzi G, Calabria A, Brasca S et al. VISPA2: a scalable pipeline for high-throughput identification and annotation of vector integration sites, BMC Bioinformatics 2017;18:520.

5. Biffi A, Montini E, Lorioli L et al. Lentiviral hematopoietic stem cell gene therapy benefits metachromatic leukodystrophy, Science (New York, N.Y.) 2013;341:1233158-1233158.

6. Aiuti A, Biasco L, Scaramuzza S et al. Lentiviral hematopoietic stem cell gene therapy in patients with Wiskott-Aldrich syndrome, Science 2013;341:1233151.
