# Supplementary material for: ISAnalytics enables longitudinal and high-throughput clonal tracking studies in hematopoietic stem cell gene therapy applications
Source: Brief Bioinform. 2022 Dec 21;24(1):bbac551. doi: 10.1093/bib/bbac551 (PMC9910212; doi:10.1093/bib/bbac551)
Supplement: Pais_et_al-ISAnalytics-Extended_Data_1-tool_comparison_bbac551 [file pais_et_al-isanalytics-extended_data_1-tool_comparison_bbac551.html]

Tool comparison: ISAnalytics and barcodetrackR


# Tool comparison: ISAnalytics and barcodetrackR

#### Giulia Pais

#### 2022-10-28

- Recalibration
  - ISAnalytics
  - barcodetrackR
- Outlier removal by pool
  - ISAnalytics
  - barcodetrackR
- Collision
  removal
  - ISAnalytics
  - barcodetrackR
- Data
  aggregation
  - barcodetrackR
- Descriptive statistics &
  diversity
  - ISAnalytics
  - barcodetrackR
    - Reshaping
      data
    - Diversity
- Abundance
  - ISAnalytics
  - barcodetrackR
- Top N clones
  - ISAnalytics
  - barcodetrackR
- Clonal counts
  - ISAnalytics
    - Default IS
      counts
    - Cumulative
      counts
  - barcodetrackR
    - Default
      IS counts
    - Cumulative counts
- Correlation and similarity
  - ISAnalytics
  - barcodetrackR
- Clonal bias
  - ISAnalytics
  - barcodetrackR
- Chord diagram
  - ISAnalytics
  - barcodetrackR
- Lineage bias analysis
  - ISAnalytics
  - barcodetrackR
- CIS statistics
  - ISAnalytics
  - barcodetrackR
- Circos genomic density
  - ISAnalytics
  - barcodetrackR
- Waves of clones over time
  - ISAnalytics
  - barcodetrackR
- Population size estimate
  - ISAnalytics
  - barcodetrackR

```
library(ISAnalytics)
```

```
## Loading required package: magrittr
```

```
library(barcodetrackR)

options(ISAnalytics.reports = FALSE)
#enable_progress_bars()
data("association_file")
data("integration_matrices")
```

# Recalibration

## ISAnalytics

```
recalibrated_data <- compute_near_integrations(integration_matrices, 
                                               map_as_file = FALSE)
head(recalibrated_data)
```

```
##    chr integration_locus strand GeneName GeneStrand
## 1:   1           8464757      -     RERE          -
## 2:   1           8464757      -     RERE          -
## 3:   1           8607362      -     RERE          -
## 4:   1           8607362      -     RERE          -
## 5:   1          12341466      -   VPS13D          +
## 6:   1          14034054      -    PRDM2          +
##                                                 CompleteAmplificationID
## 1:   PJ01_POOL01_LTR19LC2_PT001_PT001-97_lenti_GLOBE_BM_1_SLiM_0030_MNC
## 2:  PJ01_POOL01_LTR37LC2_PT001_PT001-107_lenti_GLOBE_BM_1_SLiM_0060_MNC
## 3:  PJ01_POOL01_LTR27LC94_PT001_PT001-81_lenti_GLOBE_BM_1_SLiM_0180_MNC
## 4:  PJ01_POOL01_LTR83LC66_PT001_PT001-81_lenti_GLOBE_BM_1_SLiM_0180_MNC
## 5: PJ01_POOL03_LTR93LC90_PT002_PT002-464_lenti_GLOBE_PB_1_SLiM_0360_MNC
## 6: PJ01_POOL03_LTR51LC86_PT002_PT002-466_lenti_GLOBE_BM_1_SLiM_0360_MNC
##    seqCount fragmentEstimate
## 1:      542         3.011477
## 2:        1         1.001254
## 3:     1516         3.012519
## 4:      186         1.000670
## 5:     1843         8.050805
## 6:     1938         3.008547
```

## barcodetrackR

```
### No similar functionality available
```

# Outlier removal by pool

## ISAnalytics

```
outliers_removed <- outlier_filter(association_file)
```

```
## Removing NAs from data...
## Log2 transformation, removing values <= 0
```

```
head(outliers_removed)
```

```
##                                                 CompleteAmplificationID
## 1: PJ01_POOL01_LTR75LC38_PT001_PT001-103_lenti_GLOBE_PB_1_SLiM_0060_MNC
## 2:  PJ01_POOL01_LTR53LC32_PT001_PT001-81_lenti_GLOBE_BM_1_SLiM_0180_MNC
## 3:  PJ01_POOL01_LTR83LC66_PT001_PT001-81_lenti_GLOBE_BM_1_SLiM_0180_MNC
## 4:  PJ01_POOL01_LTR27LC94_PT001_PT001-81_lenti_GLOBE_BM_1_SLiM_0180_MNC
## 5:  PJ01_POOL01_LTR69LC52_PT001_PT001-74_lenti_GLOBE_PB_1_SLiM_0180_MNC
## 6:  PJ01_POOL01_LTR37LC2_PT001_PT001-107_lenti_GLOBE_BM_1_SLiM_0060_MNC
##    ProjectID  FUSIONID PoolID TagSequence SubjectID VectorType VectorID
## 1:      PJ01 ET#382.46 POOL01   LTR75LC38     PT001      lenti    GLOBE
## 2:      PJ01 ET#381.40 POOL01   LTR53LC32     PT001      lenti    GLOBE
## 3:      PJ01  ET#381.9 POOL01   LTR83LC66     PT001      lenti    GLOBE
## 4:      PJ01 ET#381.71 POOL01   LTR27LC94     PT001      lenti    GLOBE
## 5:      PJ01  ET#381.2 POOL01   LTR69LC52     PT001      lenti    GLOBE
## 6:      PJ01 ET#382.28 POOL01    LTR37LC2     PT001      lenti    GLOBE
##    ExperimentID Tissue TimePoint DNAFragmentation PCRMethod TagIDextended
## 1:         <NA>     PB      0060            SONIC      SLiM     LTR75LC38
## 2:         <NA>     BM      0180            SONIC      SLiM     LTR53LC32
## 3:         <NA>     BM      0180            SONIC      SLiM     LTR83LC66
## 4:         <NA>     BM      0180            SONIC      SLiM     LTR27LC94
## 5:         <NA>     PB      0180            SONIC      SLiM     LTR69LC52
## 6:         <NA>     BM      0060            SONIC      SLiM      LTR37LC2
##    Keywords CellMarker      TagID NGSProvider NGSTechnology ConverrtedFilesDir
## 1:     <NA>        MNC LTR75.LC38        <NA>         HiSeq               <NA>
## 2:     <NA>        MNC LTR53.LC32        <NA>         HiSeq               <NA>
## 3:     <NA>        MNC LTR83.LC66        <NA>         HiSeq               <NA>
## 4:     <NA>        MNC LTR27.LC94        <NA>         HiSeq               <NA>
## 5:     <NA>        MNC LTR69.LC52        <NA>         HiSeq               <NA>
## 6:     <NA>        MNC  LTR37.LC2        <NA>         HiSeq               <NA>
##    ConverrtedFilesName SourceFileFolder SourceFileNameR1 SourceFileNameR2
## 1:                <NA>             <NA>             <NA>             <NA>
## 2:                <NA>             <NA>             <NA>             <NA>
## 3:                <NA>             <NA>             <NA>             <NA>
## 4:                <NA>             <NA>             <NA>             <NA>
## 5:                <NA>             <NA>             <NA>             <NA>
## 6:                <NA>             <NA>             <NA>             <NA>
##    DNAnumber ReplicateNumber DNAextractionDate DNAngUsed LinearPCRID
## 1: PT001-103               3        2016-03-16    23.184        <NA>
## 2:  PT001-81               2        2016-07-15   181.440        <NA>
## 3:  PT001-81               1        2016-07-15   181.440        <NA>
## 4:  PT001-81               3        2016-07-15   181.440        <NA>
## 5:  PT001-74               1        2016-07-15    23.058        <NA>
## 6: PT001-107               2        2016-03-16   171.360        <NA>
##    LinearPCRDate SonicationDate LigationDate 1stExpoPCRID 1stExpoPCRDate
## 1:          <NA>     2016-11-02   2016-11-02    ET#380.46     2016-11-02
## 2:          <NA>     2016-11-02   2016-11-02    ET#379.40     2016-11-02
## 3:          <NA>     2016-11-02   2016-11-02     ET#379.9     2016-11-02
## 4:          <NA>     2016-11-02   2016-11-02    ET#379.71     2016-11-02
## 5:          <NA>     2016-11-02   2016-11-02     ET#379.2     2016-11-02
## 6:          <NA>     2016-11-02   2016-11-02    ET#380.28     2016-11-02
##    2ndExpoID 2ndExpoDate FusionPrimerPCRID FusionPrimerPCRDate   PoolDate
## 1:      <NA>        <NA>         ET#382.46          2016-11-03 2016-11-07
## 2:      <NA>        <NA>         ET#381.40          2016-11-03 2016-11-07
## 3:      <NA>        <NA>          ET#381.9          2016-11-03 2016-11-07
## 4:      <NA>        <NA>         ET#381.71          2016-11-03 2016-11-07
## 5:      <NA>        <NA>          ET#381.2          2016-11-03 2016-11-07
## 6:      <NA>        <NA>         ET#382.28          2016-11-03 2016-11-07
##    SequencingDate  VCN Genome SequencingRound Genotype TestGroup  MOI
## 1:     2016-11-15 0.30   hg19               1     <NA>      <NA> <NA>
## 2:     2016-11-15 0.27   hg19               1     <NA>      <NA> <NA>
## 3:     2016-11-15 0.27   hg19               1     <NA>      <NA> <NA>
## 4:     2016-11-15 0.27   hg19               1     <NA>      <NA> <NA>
## 5:     2016-11-15 0.24   hg19               1     <NA>      <NA> <NA>
## 6:     2016-11-15 0.42   hg19               1     <NA>      <NA> <NA>
##    Engraftment Transduction Notes AddedField1 AddedField2 AddedField3
## 1:          NA           NA  <NA>        <NA>        <NA>        <NA>
## 2:          NA           NA  <NA>        <NA>        <NA>        <NA>
## 3:          NA           NA  <NA>        <NA>        <NA>        <NA>
## 4:          NA           NA  <NA>        <NA>        <NA>        <NA>
## 5:          NA           NA  <NA>        <NA>        <NA>        <NA>
## 6:          NA           NA  <NA>        <NA>        <NA>        <NA>
##    AddedField4 concatenatePoolIDSeqRun AddedField6_RelativeBloodPercentage
## 1:        <NA>                POOL01-1                                <NA>
## 2:        <NA>                POOL01-1                                <NA>
## 3:        <NA>                POOL01-1                                <NA>
## 4:        <NA>                POOL01-1                                <NA>
## 5:        <NA>                POOL01-1                                <NA>
## 6:        <NA>                POOL01-1                                <NA>
##    AddedField7_PurityTestFeasibility AddedField8_FacsSeparationPurity Kapa
## 1:                                NA                               NA   NA
## 2:                                NA                               NA   NA
## 3:                                NA                               NA   NA
## 4:                                NA                               NA   NA
## 5:                                NA                               NA   NA
## 6:                                NA                               NA   NA
##    ulForPool               UniqueID StudyTestID StudyTestGroup MouseID Tigroup
## 1:        NA ID00000000000000007433        <NA>             NA      NA    <NA>
## 2:        NA ID00000000000000007340        <NA>             NA      NA    <NA>
## 3:        NA ID00000000000000007310        <NA>             NA      NA    <NA>
## 4:        NA ID00000000000000007370        <NA>             NA      NA    <NA>
## 5:        NA ID00000000000000007303        <NA>             NA      NA    <NA>
## 6:        NA ID00000000000000007417        <NA>             NA      NA    <NA>
##    Tisource PathToFolderProjectID SamplesNameCheck TimepointDays
## 1:     <NA>                 /PJ01             <NA>          0060
## 2:     <NA>                 /PJ01             <NA>          0180
## 3:     <NA>                 /PJ01             <NA>          0180
## 4:     <NA>                 /PJ01             <NA>          0180
## 5:     <NA>                 /PJ01             <NA>          0180
## 6:     <NA>                 /PJ01             <NA>          0060
##    TimepointMonths TimepointYears ng DNA corrected      RUN_NAME PHIX_MAPPING
## 1:              02             01            23.18 PJ01|POOL01-1     43586699
## 2:              06             01           181.44 PJ01|POOL01-1     43586699
## 3:              06             01           181.44 PJ01|POOL01-1     43586699
## 4:              06             01           181.44 PJ01|POOL01-1     43586699
## 5:              06             01            23.06 PJ01|POOL01-1     43586699
## 6:              02             01           171.36 PJ01|POOL01-1     43586699
##    PLASMID_MAPPED_BYPOOL BARCODE_MUX LTR_IDENTIFIED TRIMMING_FINAL_LTRLC
## 1:               2256176      645026         645026               630965
## 2:               2256176      652208         652177               649044
## 3:               2256176      451519         451512               449669
## 4:               2256176      426500         426499               425666
## 5:               2256176       18300          18300                18290
## 6:               2256176      729327         729327               727219
##    LV_MAPPED BWA_MAPPED_OVERALL ISS_MAPPED_OVERALL RAW_READS QUALITY_PASSED
## 1:    211757             402477             219452        NA             NA
## 2:    303300             322086             222646        NA             NA
## 3:    204810             227275             149385        NA             NA
## 4:    185752             223915             143283        NA             NA
## 5:      6962              10487               5907        NA             NA
## 6:    318653             369117             235640        NA             NA
##    ISS_MAPPED_PP
## 1:            NA
## 2:            NA
## 3:            NA
## 4:            NA
## 5:            NA
## 6:            NA
```

## barcodetrackR

```
### No similar functionality available
```

# Collision removal

## ISAnalytics

```
collision_free_data <- remove_collisions(integration_matrices,
                                         outliers_removed)
```

```
## Identifying collisions...
## Processing collisions...
## Finished!
```

```
head(collision_free_data)
```

```
##    chr integration_locus strand GeneName GeneStrand
## 1:   1          16602483      +   FBXO42          -
## 2:   1          16602483      +   FBXO42          -
## 3:   1          16602483      +   FBXO42          -
## 4:   1          26446899      +   PDIK1L          +
## 5:   1          26446899      +   PDIK1L          +
## 6:   1          26446899      +   PDIK1L          +
##                                                 CompleteAmplificationID
## 1: PJ01_POOL01_LTR83LC46_PT001_PT001-107_lenti_GLOBE_BM_1_SLiM_0060_MNC
## 2:  PJ01_POOL01_LTR37LC2_PT001_PT001-107_lenti_GLOBE_BM_1_SLiM_0060_MNC
## 3: PJ01_POOL01_LTR85LC54_PT001_PT001-107_lenti_GLOBE_BM_1_SLiM_0060_MNC
## 4: PJ01_POOL01_LTR85LC54_PT001_PT001-107_lenti_GLOBE_BM_1_SLiM_0060_MNC
## 5: PJ01_POOL01_LTR83LC46_PT001_PT001-107_lenti_GLOBE_BM_1_SLiM_0060_MNC
## 6:  PJ01_POOL01_LTR69LC52_PT001_PT001-74_lenti_GLOBE_PB_1_SLiM_0180_MNC
##    seqCount fragmentEstimate
## 1:      575         5.029212
## 2:     1406         3.011178
## 3:      966         1.001441
## 4:     2623         6.046776
## 5:      636         4.016093
## 6:        3         3.009068
```

## barcodetrackR

```
### No similar functionality available
```

# Data aggregation

```
agg_key <- c("SubjectID", "CellMarker", "Tissue", "TimePoint")
agg <- aggregate_values_by_key(collision_free_data, 
                               outliers_removed, 
                               value_cols = c("seqCount", "fragmentEstimate"), 
                               key = agg_key)
agg_meta <- aggregate_metadata(outliers_removed, grouping_keys = agg_key)
head(agg)
```

```
## # A tibble: 6 × 11
##   chr   integrat…¹ strand GeneN…² GeneS…³ Subje…⁴ CellM…⁵ Tissue TimeP…⁶ seqCo…⁷
##   <chr>      <dbl> <chr>  <chr>   <chr>   <chr>   <chr>   <chr>  <chr>     <dbl>
## 1 1        8464757 -      RERE    -       PT001   MNC     BM     0030        542
## 2 1        8464757 -      RERE    -       PT001   MNC     BM     0060          1
## 3 1        8607357 +      RERE    -       PT001   MNC     BM     0060          1
## 4 1        8607357 +      RERE    -       PT001   MNC     BM     0180       1096
## 5 1        8607357 +      RERE    -       PT001   MNC     BM     0360        330
## 6 1        8607362 -      RERE    -       PT001   MNC     BM     0180       1702
## # … with 1 more variable: fragmentEstimate_sum <dbl>, and abbreviated variable
## #   names ¹​integration_locus, ²​GeneName, ³​GeneStrand, ⁴​SubjectID, ⁵​CellMarker,
## #   ⁶​TimePoint, ⁷​seqCount_sum
```

```
head(agg_meta)
```

```
## # A tibble: 6 × 19
##   SubjectID CellM…¹ Tissue TimeP…² FusionPr…³ LinearPC…⁴ VCN_avg ng DN…⁵ Kapa_…⁶
##   <chr>     <chr>   <chr>  <chr>   <date>     <date>       <dbl>   <dbl>   <dbl>
## 1 PT001     MNC     BM     0030    2016-11-03 Inf           0.26   300.      NaN
## 2 PT001     MNC     BM     0060    2016-11-03 Inf           0.42   171.      NaN
## 3 PT001     MNC     BM     0090    2016-11-03 Inf           0.35    89.2     NaN
## 4 PT001     MNC     BM     0180    2016-11-03 Inf           0.27   181.      NaN
## 5 PT001     MNC     BM     0360    2017-04-21 Inf           0.18    42       NaN
## 6 PT001     MNC     PB     0030    2016-11-03 Inf           0.23    23.8     NaN
## # … with 10 more variables: `ng DNA corrected_sum` <dbl>, ulForPool_sum <dbl>,
## #   BARCODE_MUX_sum <int>, TRIMMING_FINAL_LTRLC_sum <int>, LV_MAPPED_sum <int>,
## #   BWA_MAPPED_OVERALL_sum <int>, ISS_MAPPED_OVERALL_sum <int>,
## #   PCRMethod <chr>, NGSTechnology <chr>, DNAnumber <chr>, and abbreviated
## #   variable names ¹​CellMarker, ²​TimePoint, ³​FusionPrimerPCRDate_min,
## #   ⁴​LinearPCRDate_min, ⁵​`ng DNA corrected_avg`, ⁶​Kapa_avg
```

## barcodetrackR

```
### No similar functionality available
```

# Descriptive statistics & diversity

## ISAnalytics

```
desc_stats <- sample_statistics(agg, agg_meta,
                                sample_key = agg_key, 
                                value_columns = c("seqCount_sum", 
                                                  "fragmentEstimate_sum"))
head(desc_stats$metadata)
```

```
## # A tibble: 6 × 56
##   SubjectID CellM…¹ Tissue TimeP…² FusionPr…³ LinearPC…⁴ VCN_avg ng DN…⁵ Kapa_…⁶
##   <chr>     <chr>   <chr>  <chr>   <date>     <date>       <dbl>   <dbl>   <dbl>
## 1 PT001     MNC     BM     0030    2016-11-03 Inf           0.26   300.      NaN
## 2 PT001     MNC     BM     0060    2016-11-03 Inf           0.42   171.      NaN
## 3 PT001     MNC     BM     0090    2016-11-03 Inf           0.35    89.2     NaN
## 4 PT001     MNC     BM     0180    2016-11-03 Inf           0.27   181.      NaN
## 5 PT001     MNC     BM     0360    2017-04-21 Inf           0.18    42       NaN
## 6 PT001     MNC     PB     0030    2016-11-03 Inf           0.23    23.8     NaN
## # … with 47 more variables: `ng DNA corrected_sum` <dbl>, ulForPool_sum <dbl>,
## #   BARCODE_MUX_sum <int>, TRIMMING_FINAL_LTRLC_sum <int>, LV_MAPPED_sum <int>,
## #   BWA_MAPPED_OVERALL_sum <int>, ISS_MAPPED_OVERALL_sum <int>,
## #   PCRMethod <chr>, NGSTechnology <chr>, DNAnumber <chr>,
## #   seqCount_sum_shannon <dbl>, seqCount_sum_simpson <dbl>,
## #   seqCount_sum_invsimpson <dbl>, seqCount_sum_sum <dbl>,
## #   seqCount_sum_count <int>, fragmentEstimate_sum_shannon <dbl>, …
```

```
diversity_plot_isa <- ggplot2::ggplot(
  desc_stats$metadata,
  ggplot2::aes(x = TimePoint, y = fragmentEstimate_sum_shannon,
               group = SubjectID, color = SubjectID)
  ) +
  ggplot2::geom_point() +
  ggplot2::geom_line() +
  ggplot2::facet_wrap(~ Tissue)
diversity_plot_isa
```

## barcodetrackR

### Reshaping data

```
sparse_agg <- as_sparse_matrix(agg, seqCount = "seqCount_sum",
                               fragmentEstimate = "fragmentEstimate_sum", 
                               key = agg_key)
sparse_agg_fe <- sparse_agg$fragmentEstimate_sum %>%
  tidyr::unite(col = "id", dplyr::all_of(c(mandatory_IS_vars(), 
                                           annotation_IS_vars()))) %>%
  dplyr::mutate(dplyr::across(.cols = !.data$id,
                              .fns = ~ .x %>% tidyr::replace_na(0))) %>%
  tibble::column_to_rownames("id")
```

```
## Warning: Use of .data in tidyselect expressions was deprecated in tidyselect 1.2.0.
## ℹ Please use `"id"` instead of `.data$id`
```

```
head(sparse_agg_fe)
```

```
##                       PT001_MNC_BM_0030 PT001_MNC_BM_0060 PT001_MNC_BM_0180
## 1_8464757_-_RERE_-             3.011477          1.001254          0.000000
## 1_8607357_+_RERE_-             0.000000          1.001340          5.010689
## 1_8607362_-_RERE_-             0.000000          0.000000          4.013189
## 1_8850362_+_RERE_-             0.000000          0.000000          0.000000
## 1_11339120_+_UBIAD1_+          0.000000          8.027265          0.000000
## 1_12341466_-_VPS13D_+          0.000000          0.000000          0.000000
##                       PT001_MNC_BM_0360 PT002_MNC_BM_0360 PT001_MNC_PB_0060
## 1_8464757_-_RERE_-               0.0000          0.000000          0.000000
## 1_8607357_+_RERE_-              34.1016          0.000000          0.000000
## 1_8607362_-_RERE_-               0.0000          0.000000          0.000000
## 1_8850362_+_RERE_-               0.0000          3.009667          0.000000
## 1_11339120_+_UBIAD1_+            0.0000          0.000000          1.000363
## 1_12341466_-_VPS13D_+            0.0000          0.000000          0.000000
##                       PT001_MNC_PB_0180 PT002_MNC_PB_0360 PT002_MNC_BM_0030
## 1_8464757_-_RERE_-             0.000000          0.000000                 0
## 1_8607357_+_RERE_-             0.000000          0.000000                 0
## 1_8607362_-_RERE_-             0.000000          0.000000                 0
## 1_8850362_+_RERE_-             0.000000          0.000000                 0
## 1_11339120_+_UBIAD1_+          1.000438          0.000000                 0
## 1_12341466_-_VPS13D_+          0.000000          8.050805                 0
##                       PT002_MNC_PB_0060 PT001_MNC_PB_0030 PT002_MNC_BM_0180
## 1_8464757_-_RERE_-                    0                 0                 0
## 1_8607357_+_RERE_-                    0                 0                 0
## 1_8607362_-_RERE_-                    0                 0                 0
## 1_8850362_+_RERE_-                    0                 0                 0
## 1_11339120_+_UBIAD1_+                 0                 0                 0
## 1_12341466_-_VPS13D_+                 0                 0                 0
##                       PT001_MNC_BM_0090 PT002_MNC_BM_0060 PT002_MNC_BM_0090
## 1_8464757_-_RERE_-                    0                 0                 0
## 1_8607357_+_RERE_-                    0                 0                 0
## 1_8607362_-_RERE_-                    0                 0                 0
## 1_8850362_+_RERE_-                    0                 0                 0
## 1_11339120_+_UBIAD1_+                 0                 0                 0
## 1_12341466_-_VPS13D_+                 0                 0                 0
##                       PT002_MNC_PB_0030 PT001_MNC_PB_0090 PT001_MNC_PB_0360
## 1_8464757_-_RERE_-                    0                 0                 0
## 1_8607357_+_RERE_-                    0                 0                 0
## 1_8607362_-_RERE_-                    0                 0                 0
## 1_8850362_+_RERE_-                    0                 0                 0
## 1_11339120_+_UBIAD1_+                 0                 0                 0
## 1_12341466_-_VPS13D_+                 0                 0                 0
##                       PT002_MNC_PB_0180 PT002_MNC_PB_0090
## 1_8464757_-_RERE_-                    0                 0
## 1_8607357_+_RERE_-                    0                 0
## 1_8607362_-_RERE_-                    0                 0
## 1_8850362_+_RERE_-                    0                 0
## 1_11339120_+_UBIAD1_+                 0                 0
## 1_12341466_-_VPS13D_+                 0                 0
```

```
mod_af <- agg_meta %>%
  tidyr::unite(col = "SAMPLENAME", dplyr::all_of(agg_key), remove = FALSE)

se_bar <- create_SE(your_data = sparse_agg_fe, meta_data = mod_af)
```

```
## No threshold supplied. All barcodes will be retained. Be aware that lower abundance barcodes are likely to be less reliable due to sampling bias. To estimate an appropriate threshold, please see the barcodetrackR function `estimate_barcode_threshold`.
```

### Diversity

```
diversity_bar <- clonal_diversity(se_bar, plot_over = "TimePoint",
                                  group_by = "SubjectID", return_table = TRUE)
head(diversity_bar)
```

```
## # A tibble: 6 × 22
##   SAMPLEN…¹ Subje…² CellM…³ Tissue TimeP…⁴ FusionPr…⁵ LinearPC…⁶ VCN_avg ng.DN…⁷
##   <chr>     <chr>   <chr>   <chr>  <fct>   <date>     <date>       <dbl>   <dbl>
## 1 PT001_MN… PT001   MNC     BM     0030    2016-11-03 Inf           0.26   300. 
## 2 PT001_MN… PT001   MNC     BM     0060    2016-11-03 Inf           0.42   171. 
## 3 PT001_MN… PT001   MNC     BM     0090    2016-11-03 Inf           0.35    89.2
## 4 PT001_MN… PT001   MNC     BM     0180    2016-11-03 Inf           0.27   181. 
## 5 PT001_MN… PT001   MNC     BM     0360    2017-04-21 Inf           0.18    42  
## 6 PT001_MN… PT001   MNC     PB     0030    2016-11-03 Inf           0.23    23.8
## # … with 13 more variables: Kapa_avg <dbl>, ng.DNA.corrected_sum <dbl>,
## #   ulForPool_sum <dbl>, BARCODE_MUX_sum <int>, TRIMMING_FINAL_LTRLC_sum <int>,
## #   LV_MAPPED_sum <int>, BWA_MAPPED_OVERALL_sum <int>,
## #   ISS_MAPPED_OVERALL_sum <int>, PCRMethod <chr>, NGSTechnology <chr>,
## #   DNAnumber <chr>, index <dbl>, index_type <chr>, and abbreviated variable
## #   names ¹​SAMPLENAME, ²​SubjectID, ³​CellMarker, ⁴​TimePoint,
## #   ⁵​FusionPrimerPCRDate_min, ⁶​LinearPCRDate_min, ⁷​ng.DNA.corrected_avg
```

```
diversity_plot_bar <- clonal_diversity(se_bar, plot_over = "TimePoint",
                                       group_by = "SubjectID", 
                                       return_table = FALSE) +
  ggplot2::facet_wrap(~ Tissue)
diversity_plot_bar
```

# Abundance

## ISAnalytics

```
abundance_isa <- compute_abundance(agg, key = agg_key)
head(abundance_isa)
```

```
## # A tibble: 6 × 13
##   chr   integrat…¹ strand GeneN…² GeneS…³ Subje…⁴ CellM…⁵ Tissue TimeP…⁶ seqCo…⁷
##   <chr>      <dbl> <chr>  <chr>   <chr>   <chr>   <chr>   <chr>  <chr>     <dbl>
## 1 1        8464757 -      RERE    -       PT001   MNC     BM     0030        542
## 2 1        8464757 -      RERE    -       PT001   MNC     BM     0060          1
## 3 1        8607357 +      RERE    -       PT001   MNC     BM     0060          1
## 4 1        8607357 +      RERE    -       PT001   MNC     BM     0180       1096
## 5 1        8607357 +      RERE    -       PT001   MNC     BM     0360        330
## 6 1        8607362 -      RERE    -       PT001   MNC     BM     0180       1702
## # … with 3 more variables: fragmentEstimate_sum <dbl>,
## #   fragmentEstimate_sum_RelAbundance <dbl>,
## #   fragmentEstimate_sum_PercAbundance <dbl>, and abbreviated variable names
## #   ¹​integration_locus, ²​GeneName, ³​GeneStrand, ⁴​SubjectID, ⁵​CellMarker,
## #   ⁶​TimePoint, ⁷​seqCount_sum
```

```
alluvial_plots <- integration_alluvial_plot(abundance_isa,
                                            top_abundant_tbl = TRUE)
alluvial_plots$PT001_MNC_BM$plot
```

## barcodetrackR

```
abundance_bar <- rank_abundance_plot(se_bar, return_table = TRUE)
head(abundance_bar)
```

```
## # A tibble: 6 × 5
##   sample_name       percentage cumulative_sum  rank scaled_rank
##   <fct>                  <dbl>          <dbl> <int>       <dbl>
## 1 PT001_MNC_BM_0030     0.0793         0.0793     1      0     
## 2 PT001_MNC_BM_0030     0.0704         0.150      2      0.0189
## 3 PT001_MNC_BM_0030     0.0527         0.202      3      0.0377
## 4 PT001_MNC_BM_0030     0.0439         0.246      4      0.0566
## 5 PT001_MNC_BM_0030     0.0438         0.290      5      0.0755
## 6 PT001_MNC_BM_0030     0.0351         0.325      6      0.0943
```

```
abundance_bar_plot <- rank_abundance_plot(se_bar, return_table = FALSE)
abundance_bar_plot
```

```
try({
  clonal_contrib <- clonal_contribution(your_SE = se_bar, 
                                        SAMPLENAME_choice = "PT001_MNC_BM_0030", 
                                        plot_over = "TimePoint", 
                                        filter_by = "Tissue",
                                        filter_selection = "BM", 
                                        graph_type = "bar",
                                        n_clones = 10)
})
```

```
## Duplicate samples with the same value of the plot_over variable: TimePoint 
## TimePoint value = 0030 ; Duplicate sample names = PT001_MNC_BM_0030 PT002_MNC_BM_0030 
## Error in `[.data.frame`(your_data, , duplicated_samplenames) : 
##   undefined columns selected
```

# Top N clones

## ISAnalytics

```
top_10_ab_clones_isa <- top_integrations(abundance_isa, n = 10, key = agg_key)
gridExtra::grid.arrange(alluvial_plots$PT001_MNC_BM$tables)
```

## barcodetrackR

```
top_10_clones_bar <- barcode_ggheatmap(se_bar, n_clones = 10, 
                                       return_table = TRUE)
head(top_10_clones_bar)
```

```
## # A tibble: 6 × 4
##   sequence              sample_name       value cellnote
##   <fct>                 <fct>             <dbl> <chr>   
## 1 1_12341466_-_VPS13D_+ PT001_MNC_BM_0030     0 <NA>    
## 2 1_12341466_-_VPS13D_+ PT001_MNC_BM_0060     0 <NA>    
## 3 1_12341466_-_VPS13D_+ PT001_MNC_BM_0180     0 <NA>    
## 4 1_12341466_-_VPS13D_+ PT001_MNC_BM_0360     0 <NA>    
## 5 1_12341466_-_VPS13D_+ PT002_MNC_BM_0360     0 <NA>    
## 6 1_12341466_-_VPS13D_+ PT001_MNC_PB_0060     0 <NA>
```

```
top_10_clones_bar_plot <- barcode_ggheatmap(se_bar, n_clones = 10, 
                                            return_table = FALSE)
top_10_clones_bar_plot
```

# Clonal counts

## ISAnalytics

### Default IS counts

```
clonal_count_isa_plot <- ggplot2::ggplot(desc_stats$metadata,
                                         ggplot2::aes(
                                           x = TimePoint,
                                           y = nIS,
                                           group = SubjectID,
                                           color = SubjectID
                                         )) +
  ggplot2::geom_point(size = 3) +
  ggplot2::geom_line(size = 2) +
  ggplot2::facet_wrap(~Tissue) +
  ggplot2::theme_bw()
clonal_count_isa_plot
```

### Cumulative counts

```
cumulative_is_isa <- cumulative_is(agg, key = agg_key)
head(cumulative_is_isa)
```

```
## $coordinates
##       SubjectID CellMarker Tissue TimePoint chr integration_locus strand
##    1:     PT001        MNC     BM        30   1           8464757      -
##    2:     PT001        MNC     BM        30   1          16186297      -
##    3:     PT001        MNC     BM        30   1          40689188      +
##    4:     PT001        MNC     BM        30   1         157759338      -
##    5:     PT001        MNC     BM        30   1         234596545      -
##   ---                                                                   
## 2348:     PT002        MNC     PB       360   5         176653985      +
## 2349:     PT002        MNC     PB       360   6         147673451      -
## 2350:     PT002        MNC     PB       360   7           5039809      -
## 2351:     PT002        MNC     PB       360   7          17227687      +
## 2352:     PT002        MNC     PB       360   9         127351995      -
##       GeneName GeneStrand
##    1:     RERE          -
##    2:     SPEN          +
##    3:      RLF          +
##    4:    FCRL1          -
##    5:   TARBP1          -
##   ---                    
## 2348:     NSD1          +
## 2349:   STXBP5          +
## 2350: RNF216P1          +
## 2351:      AHR          +
## 2352:    NR6A1          -
## 
## $counts
##     SubjectID CellMarker Tissue TimePoint is_n_cumulative
##  1:     PT001        MNC     BM        30              54
##  2:     PT001        MNC     BM        60             147
##  3:     PT001        MNC     BM        90             179
##  4:     PT001        MNC     BM       180             240
##  5:     PT001        MNC     BM       360             240
##  6:     PT001        MNC     PB        30              28
##  7:     PT001        MNC     PB        60              77
##  8:     PT001        MNC     PB        90             104
##  9:     PT001        MNC     PB       180             121
## 10:     PT001        MNC     PB       360             121
## 11:     PT002        MNC     BM        30              97
## 12:     PT002        MNC     BM        60             124
## 13:     PT002        MNC     BM        90             139
## 14:     PT002        MNC     BM       180             181
## 15:     PT002        MNC     BM       360             260
## 16:     PT002        MNC     PB        30              14
## 17:     PT002        MNC     PB        60              25
## 18:     PT002        MNC     PB        90              37
## 19:     PT002        MNC     PB       180              60
## 20:     PT002        MNC     PB       360             104
```

```
cumulative_is_isa_plot <- ggplot2::ggplot(cumulative_is_isa$counts,
                                          ggplot2::aes(
                                            x = TimePoint,
                                            y = is_n_cumulative,
                                            group = SubjectID,
                                            color = SubjectID
                                          )) +
  ggplot2::geom_point(size = 3) +
  ggplot2::geom_line(size = 2) +
  ggplot2::facet_wrap(~Tissue) +
  ggplot2::theme_bw()
cumulative_is_isa_plot
```

## barcodetrackR

### Default IS counts

```
clonal_count_bar <- clonal_count(se_bar, 
                                 plot_over = "TimePoint",
                                 group_by = "SubjectID") +
  ggplot2::facet_wrap(~Tissue)
clonal_count_bar
```

### Cumulative counts

```
cumulative_is_bar <- clonal_count(se_bar, 
                                  plot_over = "TimePoint",
                                  group_by = "SubjectID",
                                  #group_by = c("SubjectID", "Tissue"), 
                                  cumulative = TRUE) +
  ggplot2::facet_wrap(~Tissue)
cumulative_is_bar
```

# Correlation and similarity

## ISAnalytics

```
### No similar functionality available
```

## barcodetrackR

```
sample_1 <- c("PT001_MNC_BM_0030", "PT001_MNC_PB_0030")
corr_scatter <- scatter_plot(se_bar[, sample_1], 
                             your_title = "PT001, 30 days, BM vs. PB")
corr_scatter
```

```
corr_hm <- cor_plot(se_bar, method_corr = "pearson", 
                    plot_type = "color")
corr_hm
```

```
dist_hm <- dist_plot(se_bar, plot_type = "color", assay = "counts")
dist_hm
```

# Clonal bias

## ISAnalytics

```
### No similar functionality available
```

## barcodetrackR

```
se_bar$Subject_Tissue <- paste0(se_bar$SubjectID, "_", se_bar$Tissue)
try({
  bias_plot <- bias_histogram(se_bar, 
                              split_bias_on = "Subject_Tissue",
                              bias_1 = "PT001_BM",
                              bias_2 = "PT001_PB",
                              split_bias_over = "TimePoint")
})
```

```
## Warning in max(unlist(lapply(plot_list, function(x) {: no non-missing arguments
## to max; returning -Inf
```

```
## Error in grobs[[i]] : subscript out of bounds
```

```
bias_ridge <- bias_ridge_plot(se_bar, 
                              split_bias_on = "Subject_Tissue",
                              bias_1 = "PT001_BM",
                              bias_2 = "PT001_PB",
                              split_bias_over = "TimePoint")
bias_ridge
```

```
try({
  bias_line <- bias_lineplot(
    se_bar, 
    split_bias_on = "Subject_Tissue",
    bias_1 = "PT001_BM",
    bias_2 = "PT001_PB",
    split_bias_over = "TimePoint"
  )
})
```

```
## Error in UseMethod("group_by") : 
##   no applicable method for 'group_by' applied to an object of class "NULL"
```

# Chord diagram

## ISAnalytics

```
### No similar functionality available
```

## barcodetrackR

```
try({
  ch_diag <- chord_diagram(se_bar[, sample_1], plot_label = "Tissue")
})
```

```
## Error in `levels<-`(`*tmp*`, value = as.character(levels)) : 
##   factor level [2] is duplicated
```

# Lineage bias analysis

## ISAnalytics

```
sharing <- is_sharing(agg,
                      minimal = FALSE,
                      include_self_comp = TRUE, 
                      table_for_venn = TRUE
)
```

```
## Calculating combinations...
## Calculating self groups (requested)...
## Calculating permutations (requested)...
## Done!
```

```
head(sharing)
```

```
##                   g1                g2 shared count_g1 count_g2 count_union
## 1: PT001_MNC_BM_0030 PT001_MNC_BM_0030     54       54       54          54
## 2: PT001_MNC_BM_0030 PT001_MNC_BM_0060     21       54      114         147
## 3: PT001_MNC_BM_0060 PT001_MNC_BM_0030     21      114       54         147
## 4: PT001_MNC_BM_0060 PT001_MNC_BM_0060    114      114      114         114
## 5: PT001_MNC_BM_0030 PT001_MNC_BM_0180     15       54       89         128
## 6: PT001_MNC_BM_0180 PT001_MNC_BM_0030     15       89       54         128
##        on_g1     on_g2  on_union      truth_tbl_venn
## 1: 100.00000 100.00000 100.00000  <data.table[54x2]>
## 2:  38.88889  18.42105  14.28571 <data.table[147x3]>
## 3:  18.42105  38.88889  14.28571 <data.table[147x3]>
## 4: 100.00000 100.00000 100.00000 <data.table[114x2]>
## 5:  27.77778  16.85393  11.71875 <data.table[128x3]>
## 6:  16.85393  27.77778  11.71875 <data.table[128x3]>
```

```
sharing_heatmaps <- sharing_heatmap(sharing_df = sharing)
sharing_heatmaps$absolute
```

```
venn_tbls <- sharing_venn(sharing, row_range = 2:3, euler = FALSE)
plot(venn_tbls[[1]], quantities = TRUE)
```

## barcodetrackR

```
### No similar functionality available
```

# CIS statistics

## ISAnalytics

```
cis <- CIS_grubbs(agg, by = "SubjectID", results_as_list = FALSE)
```

```
## Warning: Warning: missing genes in refgenes table
## ℹ A total of 5 genes were found in the input data but not in the refgene table. This may be caused by a mismatch in the annotation phase of the matrix. Here is a summary: 
## # A tibble: 5 × 3
##   chr   GeneName  GeneStrand
##   <chr> <chr>     <chr>     
## 1 14    PLEKHG4B  -         
## 2 15    CRELD2    -         
## 3 16    UBE2D2    +         
## 4 19    LINC01133 +         
## 5 6     HTR4      +         
## ℹ NOTE: missing genes will be removed from the final output! Review results carefully
## ℹ A total of 25 IS will be removed because of missing genes ( 2.35 % of total IS in input)
```

```
head(cis$cis)
```

```
## # A tibble: 6 × 38
##   GeneName GeneStr…¹ chr       n   mean    sd median trimmed   mad    min    max
##   <chr>    <chr>     <chr> <int>  <dbl> <dbl>  <dbl>   <dbl> <dbl>  <dbl>  <dbl>
## 1 ABHD2    +         15        2 8.96e7     0 8.96e7  8.96e7     0 8.96e7 8.96e7
## 2 ACAP2    -         3         2 1.95e8     0 1.95e8  1.95e8     0 1.95e8 1.95e8
## 3 ACOX1    -         17        3 7.40e7     0 7.40e7  7.40e7     0 7.40e7 7.40e7
## 4 ADD1     +         4         3 2.86e6     0 2.86e6  2.86e6     0 2.86e6 2.86e6
## 5 ADGRA3   -         4         2 2.25e7     0 2.25e7  2.25e7     0 2.25e7 2.25e7
## 6 ADGRB3   +         6         2 6.94e7     0 6.94e7  6.94e7     0 6.94e7 6.94e7
## # … with 27 more variables: range <dbl>, skew <dbl>, kurtosis <dbl>,
## #   n_IS_perGene <int>, min_bp_integration_locus <dbl>,
## #   max_bp_integration_locus <dbl>, IS_span_bp <dbl>,
## #   avg_bp_integration_locus <dbl>, median_bp_integration_locus <dbl>,
## #   distinct_orientations <int>, average_TxLen <dbl>,
## #   raw_gene_integration_frequency <dbl>,
## #   integration_frequency_withtolerance <dbl>, …
```

```
cis_plot <- CIS_volcano_plot(cis$cis) +
  ggplot2::facet_wrap(~group)
```

```
## Loading annotated genes -  species selected: 
## • Homo sapiens (Human)
## Loading annotated genes -  done
```

```
cis_plot
```

```
cis_overtime <- CIS_grubbs_overtime(agg)
```

```
## Warning: Warning: missing genes in refgenes table
## ℹ A total of 5 genes were found in the input data but not in the refgene table. This may be caused by a mismatch in the annotation phase of the matrix. Here is a summary: 
## # A tibble: 5 × 3
##   chr   GeneName  GeneStrand
##   <chr> <chr>     <chr>     
## 1 14    PLEKHG4B  -         
## 2 15    CRELD2    -         
## 3 16    UBE2D2    +         
## 4 19    LINC01133 +         
## 5 6     HTR4      +         
## ℹ NOTE: missing genes will be removed from the final output! Review results carefully
## ℹ A total of 25 IS will be removed because of missing genes ( 2.35 % of total IS in input)
```

```
cis_overtime_hmaps <- top_cis_overtime_heatmap(cis_overtime$cis,
                                  fill_NA_in_heatmap = TRUE
)
```

```
## Loading annotated genes -  species selected: 
## • Homo sapiens (Human)
## Loading annotated genes -  done
```

## barcodetrackR

```
### No similar functionality available
```

# Circos genomic density

## ISAnalytics

```
by_subj <- agg %>%
  dplyr::group_by(.data$SubjectID) %>%
  dplyr::group_split()
circos_genomic_density(by_subj,
                       track_colors = c("navyblue", "gold"),
                       grDevice = "default", track.height = 0.1
)
```

## barcodetrackR

```
### No similar functionality available
```

# Waves of clones over time

## ISAnalytics

```
df1 <- agg %>%
  dplyr::filter(.data$Tissue == "BM")
df2 <- agg %>%
  dplyr::filter(.data$Tissue == "PB")
source <- iss_source(df1, df2)
head(source)
```

```
## $PT001
## # A tibble: 161 × 14
##    g1      g1_Su…¹ g1_Ce…² g1_Ti…³ g1_Ti…⁴ g2    g2_Su…⁵ g2_Ce…⁶ g2_Ti…⁷ g2_Ti…⁸
##    <chr>   <chr>   <chr>   <chr>     <int> <chr> <chr>   <chr>   <chr>     <int>
##  1 PT001_… PT001   MNC     BM           30 PT00… PT001   MNC     PB           60
##  2 PT001_… PT001   MNC     BM           30 PT00… PT001   MNC     PB           60
##  3 PT001_… PT001   MNC     BM           30 PT00… PT001   MNC     PB           60
##  4 PT001_… PT001   MNC     BM           30 PT00… PT001   MNC     PB           60
##  5 PT001_… PT001   MNC     BM           30 PT00… PT001   MNC     PB           60
##  6 PT001_… PT001   MNC     BM           30 PT00… PT001   MNC     PB           60
##  7 PT001_… PT001   MNC     BM           30 PT00… PT001   MNC     PB           60
##  8 PT001_… PT001   MNC     BM           30 PT00… PT001   MNC     PB           60
##  9 PT001_… PT001   MNC     BM           60 PT00… PT001   MNC     PB           60
## 10 PT001_… PT001   MNC     BM           60 PT00… PT001   MNC     PB           60
## # … with 151 more rows, 4 more variables: chr <chr>, integration_locus <dbl>,
## #   strand <chr>, sharing_perc <dbl>, and abbreviated variable names
## #   ¹​g1_SubjectID, ²​g1_CellMarker, ³​g1_Tissue, ⁴​g1_TimePoint, ⁵​g2_SubjectID,
## #   ⁶​g2_CellMarker, ⁷​g2_Tissue, ⁸​g2_TimePoint
## 
## $PT002
## # A tibble: 77 × 14
##    g1      g1_Su…¹ g1_Ce…² g1_Ti…³ g1_Ti…⁴ g2    g2_Su…⁵ g2_Ce…⁶ g2_Ti…⁷ g2_Ti…⁸
##    <chr>   <chr>   <chr>   <chr>     <int> <chr> <chr>   <chr>   <chr>     <int>
##  1 PT002_… PT002   MNC     BM          360 PT00… PT002   MNC     PB          360
##  2 PT002_… PT002   MNC     BM          360 PT00… PT002   MNC     PB          360
##  3 PT002_… PT002   MNC     BM           30 PT00… PT002   MNC     PB          360
##  4 PT002_… PT002   MNC     BM          180 PT00… PT002   MNC     PB          360
##  5 PT002_… PT002   MNC     BM          180 PT00… PT002   MNC     PB          360
##  6 PT002_… PT002   MNC     BM           60 PT00… PT002   MNC     PB          360
##  7 PT002_… PT002   MNC     BM           90 PT00… PT002   MNC     PB          360
##  8 PT002_… PT002   MNC     BM          360 PT00… PT002   MNC     PB           60
##  9 PT002_… PT002   MNC     BM           30 PT00… PT002   MNC     PB           60
## 10 PT002_… PT002   MNC     BM           30 PT00… PT002   MNC     PB           60
## # … with 67 more rows, 4 more variables: chr <chr>, integration_locus <dbl>,
## #   strand <chr>, sharing_perc <dbl>, and abbreviated variable names
## #   ¹​g1_SubjectID, ²​g1_CellMarker, ³​g1_Tissue, ⁴​g1_TimePoint, ⁵​g2_SubjectID,
## #   ⁶​g2_CellMarker, ⁷​g2_Tissue, ⁸​g2_TimePoint
```

```
iss_source_plot <- ggplot2::ggplot(source$PT001, ggplot2::aes(
  x = as.factor(g2_TimePoint),
  y = sharing_perc, fill = g1
)) +
  ggplot2::geom_col() +
  ggplot2::labs(
    x = "Time point", y = "Shared IS % with MNC BM",
    title = "Source of is MNC BM vs MNC PB"
  ) +
  ggplot2::theme_bw()
iss_source_plot
```

## barcodetrackR

```
### No similar functionality available
```

# Population size estimate

## ISAnalytics

```
estimate <- HSC_population_size_estimate(
  x = agg,
  metadata = agg_meta,
  fragmentEstimate_column = "fragmentEstimate_sum",
  stable_timepoints = c(90, 180, 360),
  cell_type = "Other"
)
```

```
## Calculating number of IS for each group...
```

```
head(estimate$est)
```

```
## # A tibble: 6 × 12
##   Model    abund…¹ stderr Subje…² Timep…³ CellT…⁴ Tissue TimeP…⁵ TimeP…⁶ Model…⁷
##   <chr>      <dbl>  <dbl> <chr>   <chr>   <chr>   <chr>    <dbl>   <dbl> <chr>  
## 1 M0         152.    8.65 PT001   All     Other   PB          30     360 Closed…
## 2 Mh Chao…   159.   13.1  PT001   All     Other   PB          30     360 Closed…
## 3 Mh Pois…   157.   12.2  PT001   All     Other   PB          30     360 Closed…
## 4 Mh Darr…   166.   21.0  PT001   All     Other   PB          30     360 Closed…
## 5 Mh Gamm…   175.   33.2  PT001   All     Other   PB          30     360 Closed…
## 6 M0          88.7   5.86 PT001   Stable  Other   PB          90     360 Closed…
## # … with 2 more variables: ModelSetUp <chr>, PopSize <dbl>, and abbreviated
## #   variable names ¹​abundance, ²​SubjectID, ³​Timepoints, ⁴​CellType,
## #   ⁵​TimePoint_from, ⁶​TimePoint_to, ⁷​ModelType
```

```
estimate_plot <- HSC_population_plot(estimate$est, "PJ01")
estimate_plot
```

## barcodetrackR

```
### No similar functionality available
```
