# Supplementary material for: ISAnalytics enables longitudinal and high-throughput clonal tracking studies in hematopoietic stem cell gene therapy applications
Source: Brief Bioinform. 2022 Dec 21;24(1):bbac551. doi: 10.1093/bib/bbac551 (PMC9910212; doi:10.1093/bib/bbac551)
Supplement: Pais_et_al-ISAnalytics-Extended_Data_2-barcoding_bbac551 [file pais_et_al-isanalytics-extended_data_2-barcoding_bbac551.html]

ISAnalytics use case: barcodes data


# ISAnalytics use case: barcodes data

#### Giulia Pais

#### 2022-10-25

```
library(ISAnalytics)
```

```
## Loading required package: magrittr
```

```
# Set appropriate data and metadata specs ----
metadata_specs <- tibble::tribble(
  ~names, ~types, ~transform, ~flag, ~tag,
  "ProjectID", "char", NULL, "required", "project_id",
  "SubjectID", "char", NULL, "required", "subject",
  "Tissue", "char", NULL, "required", "tissue",
  "TimePoint", "int", NULL, "required", "tp_days",
  "CellMarker", "char", NULL, "required", "cell_marker",
  "ID", "char", NULL, "required", "pcr_repl_id",
  "SourceFileName", "char", NULL, "optional", NA_character_,
  "Link", "char", NULL, "optional", NA_character_
)
set_af_columns_def(metadata_specs)
```

```
## Warning: Warning: important tags missing
## ℹ Some tags are required for proper execution of some functions. If these tags are not provided, execution of dependent functions might fail. Review your inputs carefully.
## ℹ Missing tags: pool_id, fusion_id, tag_seq, vector_id, tag_id, pcr_replicate, vispa_concatenate, proj_folder
## ℹ To see where these are involved type `inspect_tags(c('pool_id','fusion_id','tag_seq','vector_id','tag_id','pcr_replicate','vispa_concatenate','proj_folder'))`
```

```
## Association file columns specs successfully changed
```

```
mandatory_specs <- tibble::tribble(
  ~names, ~types, ~transform, ~flag, ~tag,
  "BarcodeSeq", "char", NULL, "required", NA_character_
)
set_mandatory_IS_vars(mandatory_specs)
```

```
## Warning: Warning: important tags missing
## ℹ Some tags are required for proper execution of some functions. If these tags are not provided, execution of dependent functions might fail. Review your inputs carefully.
## ℹ Missing tags: chromosome, locus, is_strand
## ℹ To see where these are involved type `inspect_tags(c('chromosome','locus','is_strand'))`
```

```
## Mandatory IS vars successfully changed
```

```
# Files ----
data_folder <- "."
meta_file <- "BarcodesPMID32601433.xlsx"
matrix_file <- "GSE144340_Matrix_542.tsv"

# Data import ----
af <- import_association_file(fs::path(data_folder, meta_file),
                              report_path = NULL)
```

```
## Warning: file in xls/xlsx format
## ℹ The use of xls/xlsx is discouraged as it can lead to potential problems in parsing data. Use of *.tsv or *.csv is recommended.
## Carefully review the data after importing!
```

```
## 
-
/
                                                                              

/
                                                                              

-
```

```
af
```

```
##        ProjectID SubjectID Tissue TimePoint CellMarker      ID
##  1: PMID32601433        A0     BM        21      Whole   BM_A0
##  2: PMID32601433        A0     PB        21      Whole PB21_A0
##  3: PMID32601433        A1     BM        21      Whole   BM_A1
##  4: PMID32601433        A1     PB        21      Whole PB21_A1
##  5: PMID32601433        A2     PB        21      Whole PB21_A2
##  6: PMID32601433        A3     PB        21      Whole PB21_A3
##  7: PMID32601433        A4     BM        21      Whole   BM_A4
##  8: PMID32601433        A4     PB        21      Whole PB21_A4
##  9: PMID32601433        C0     PB        21      Whole PB21_C0
## 10: PMID32601433        C1     BM        21      Whole   BM_C1
## 11: PMID32601433        C1     PB        21      Whole PB21_C1
## 12: PMID32601433        C2     BM        21      Whole   BM_C2
## 13: PMID32601433        C2     PB        21      Whole PB21_C2
## 14: PMID32601433        C3     BM        21      Whole   BM_C3
## 15: PMID32601433        C3     PB        21      Whole PB21_C3
##                  SourceFileName
##  1: GSE144340_Matrix_542.tsv.gz
##  2: GSE144340_Matrix_542.tsv.gz
##  3: GSE144340_Matrix_542.tsv.gz
##  4: GSE144340_Matrix_542.tsv.gz
##  5: GSE144340_Matrix_542.tsv.gz
##  6: GSE144340_Matrix_542.tsv.gz
##  7: GSE144340_Matrix_542.tsv.gz
##  8: GSE144340_Matrix_542.tsv.gz
##  9: GSE144340_Matrix_542.tsv.gz
## 10: GSE144340_Matrix_542.tsv.gz
## 11: GSE144340_Matrix_542.tsv.gz
## 12: GSE144340_Matrix_542.tsv.gz
## 13: GSE144340_Matrix_542.tsv.gz
## 14: GSE144340_Matrix_542.tsv.gz
## 15: GSE144340_Matrix_542.tsv.gz
##                                                                                                      Link
##  1: https://ftp.ncbi.nlm.nih.gov/geo/series/GSE144nnn/GSE144340/suppl/GSE144340%5FMatrix%5F542%2Etsv%2Egz
##  2: https://ftp.ncbi.nlm.nih.gov/geo/series/GSE144nnn/GSE144340/suppl/GSE144340%5FMatrix%5F542%2Etsv%2Egz
##  3: https://ftp.ncbi.nlm.nih.gov/geo/series/GSE144nnn/GSE144340/suppl/GSE144340%5FMatrix%5F542%2Etsv%2Egz
##  4: https://ftp.ncbi.nlm.nih.gov/geo/series/GSE144nnn/GSE144340/suppl/GSE144340%5FMatrix%5F542%2Etsv%2Egz
##  5: https://ftp.ncbi.nlm.nih.gov/geo/series/GSE144nnn/GSE144340/suppl/GSE144340%5FMatrix%5F542%2Etsv%2Egz
##  6: https://ftp.ncbi.nlm.nih.gov/geo/series/GSE144nnn/GSE144340/suppl/GSE144340%5FMatrix%5F542%2Etsv%2Egz
##  7: https://ftp.ncbi.nlm.nih.gov/geo/series/GSE144nnn/GSE144340/suppl/GSE144340%5FMatrix%5F542%2Etsv%2Egz
##  8: https://ftp.ncbi.nlm.nih.gov/geo/series/GSE144nnn/GSE144340/suppl/GSE144340%5FMatrix%5F542%2Etsv%2Egz
##  9: https://ftp.ncbi.nlm.nih.gov/geo/series/GSE144nnn/GSE144340/suppl/GSE144340%5FMatrix%5F542%2Etsv%2Egz
## 10: https://ftp.ncbi.nlm.nih.gov/geo/series/GSE144nnn/GSE144340/suppl/GSE144340%5FMatrix%5F542%2Etsv%2Egz
## 11: https://ftp.ncbi.nlm.nih.gov/geo/series/GSE144nnn/GSE144340/suppl/GSE144340%5FMatrix%5F542%2Etsv%2Egz
## 12: https://ftp.ncbi.nlm.nih.gov/geo/series/GSE144nnn/GSE144340/suppl/GSE144340%5FMatrix%5F542%2Etsv%2Egz
## 13: https://ftp.ncbi.nlm.nih.gov/geo/series/GSE144nnn/GSE144340/suppl/GSE144340%5FMatrix%5F542%2Etsv%2Egz
## 14: https://ftp.ncbi.nlm.nih.gov/geo/series/GSE144nnn/GSE144340/suppl/GSE144340%5FMatrix%5F542%2Etsv%2Egz
## 15: https://ftp.ncbi.nlm.nih.gov/geo/series/GSE144nnn/GSE144340/suppl/GSE144340%5FMatrix%5F542%2Etsv%2Egz
##     TimepointMonths TimepointYears
##  1:              01             01
##  2:              01             01
##  3:              01             01
##  4:              01             01
##  5:              01             01
##  6:              01             01
##  7:              01             01
##  8:              01             01
##  9:              01             01
## 10:              01             01
## 11:              01             01
## 12:              01             01
## 13:              01             01
## 14:              01             01
## 15:              01             01
```

```
matrix <- import_single_Vispa2Matrix(fs::path(data_folder, matrix_file),
                                     sample_names_to = "ID")
```

```
## Reading file...
## ℹ Mode: fread
```

```
## Reshaping...
## *** File info *** 
## • --- Annotated: FALSE
## • --- Dimensions: 31757 x 16
## • --- Read mode: fread
## • --- Sample count: 15
```

```
matrix
```

```
##                    BarcodeSeq      ID Value
##     1: AAAAAAAATTTTTAAACGTACC   BM_A0     1
##     2: AAAAAACATATCTATAGTTACC   BM_A0     1
##     3: AAAAAATATATAAATAGATACC   BM_A0     1
##     4: AAAAACAACAAGGAAATTCAAT   BM_A0     1
##     5: AAAAACAACGAGGATAGTGAAT   BM_A0     1
##    ---                                     
## 33772: TTTTGAGACCTTCACACCTACT PB21_C3     1
## 33773: TTTTGCCACCTTCATACCCAAC PB21_C3     1
## 33774: TTTTTAAACCGTTAGACCCGCA PB21_C3     1
## 33775: TTTTTTCACGACAATAGCCAAT PB21_C3     1
## 33776: TTTTTTCACTTGCACATCCGGC PB21_C3     1
```

```
matrix %>% 
  readr::write_tsv(file = "GSE144340_Matrix_542_tidy.tsv")


# Descriptive stats ----
desc_stats <- sample_statistics(matrix, af,
                                sample_key = pcr_id_column(),
                                value_columns = "Value")$metadata %>%
  dplyr::rename(distinct_barcodes = "nIS")
desc_stats
```

```
##        ProjectID SubjectID Tissue TimePoint CellMarker      ID
##  1: PMID32601433        A0     BM        21      Whole   BM_A0
##  2: PMID32601433        A0     PB        21      Whole PB21_A0
##  3: PMID32601433        A1     BM        21      Whole   BM_A1
##  4: PMID32601433        A1     PB        21      Whole PB21_A1
##  5: PMID32601433        A2     PB        21      Whole PB21_A2
##  6: PMID32601433        A3     PB        21      Whole PB21_A3
##  7: PMID32601433        A4     BM        21      Whole   BM_A4
##  8: PMID32601433        A4     PB        21      Whole PB21_A4
##  9: PMID32601433        C0     PB        21      Whole PB21_C0
## 10: PMID32601433        C1     BM        21      Whole   BM_C1
## 11: PMID32601433        C1     PB        21      Whole PB21_C1
## 12: PMID32601433        C2     BM        21      Whole   BM_C2
## 13: PMID32601433        C2     PB        21      Whole PB21_C2
## 14: PMID32601433        C3     BM        21      Whole   BM_C3
## 15: PMID32601433        C3     PB        21      Whole PB21_C3
##                  SourceFileName
##  1: GSE144340_Matrix_542.tsv.gz
##  2: GSE144340_Matrix_542.tsv.gz
##  3: GSE144340_Matrix_542.tsv.gz
##  4: GSE144340_Matrix_542.tsv.gz
##  5: GSE144340_Matrix_542.tsv.gz
##  6: GSE144340_Matrix_542.tsv.gz
##  7: GSE144340_Matrix_542.tsv.gz
##  8: GSE144340_Matrix_542.tsv.gz
##  9: GSE144340_Matrix_542.tsv.gz
## 10: GSE144340_Matrix_542.tsv.gz
## 11: GSE144340_Matrix_542.tsv.gz
## 12: GSE144340_Matrix_542.tsv.gz
## 13: GSE144340_Matrix_542.tsv.gz
## 14: GSE144340_Matrix_542.tsv.gz
## 15: GSE144340_Matrix_542.tsv.gz
##                                                                                                      Link
##  1: https://ftp.ncbi.nlm.nih.gov/geo/series/GSE144nnn/GSE144340/suppl/GSE144340%5FMatrix%5F542%2Etsv%2Egz
##  2: https://ftp.ncbi.nlm.nih.gov/geo/series/GSE144nnn/GSE144340/suppl/GSE144340%5FMatrix%5F542%2Etsv%2Egz
##  3: https://ftp.ncbi.nlm.nih.gov/geo/series/GSE144nnn/GSE144340/suppl/GSE144340%5FMatrix%5F542%2Etsv%2Egz
##  4: https://ftp.ncbi.nlm.nih.gov/geo/series/GSE144nnn/GSE144340/suppl/GSE144340%5FMatrix%5F542%2Etsv%2Egz
##  5: https://ftp.ncbi.nlm.nih.gov/geo/series/GSE144nnn/GSE144340/suppl/GSE144340%5FMatrix%5F542%2Etsv%2Egz
##  6: https://ftp.ncbi.nlm.nih.gov/geo/series/GSE144nnn/GSE144340/suppl/GSE144340%5FMatrix%5F542%2Etsv%2Egz
##  7: https://ftp.ncbi.nlm.nih.gov/geo/series/GSE144nnn/GSE144340/suppl/GSE144340%5FMatrix%5F542%2Etsv%2Egz
##  8: https://ftp.ncbi.nlm.nih.gov/geo/series/GSE144nnn/GSE144340/suppl/GSE144340%5FMatrix%5F542%2Etsv%2Egz
##  9: https://ftp.ncbi.nlm.nih.gov/geo/series/GSE144nnn/GSE144340/suppl/GSE144340%5FMatrix%5F542%2Etsv%2Egz
## 10: https://ftp.ncbi.nlm.nih.gov/geo/series/GSE144nnn/GSE144340/suppl/GSE144340%5FMatrix%5F542%2Etsv%2Egz
## 11: https://ftp.ncbi.nlm.nih.gov/geo/series/GSE144nnn/GSE144340/suppl/GSE144340%5FMatrix%5F542%2Etsv%2Egz
## 12: https://ftp.ncbi.nlm.nih.gov/geo/series/GSE144nnn/GSE144340/suppl/GSE144340%5FMatrix%5F542%2Etsv%2Egz
## 13: https://ftp.ncbi.nlm.nih.gov/geo/series/GSE144nnn/GSE144340/suppl/GSE144340%5FMatrix%5F542%2Etsv%2Egz
## 14: https://ftp.ncbi.nlm.nih.gov/geo/series/GSE144nnn/GSE144340/suppl/GSE144340%5FMatrix%5F542%2Etsv%2Egz
## 15: https://ftp.ncbi.nlm.nih.gov/geo/series/GSE144nnn/GSE144340/suppl/GSE144340%5FMatrix%5F542%2Etsv%2Egz
##     TimepointMonths TimepointYears Value_shannon Value_simpson Value_invsimpson
##  1:              01             01      2.952237     0.9113968        11.286277
##  2:              01             01      3.459660     0.9488818        19.562506
##  3:              01             01      3.006200     0.8870954         8.857038
##  4:              01             01      3.774526     0.9453922        18.312391
##  5:              01             01      3.181671     0.9222549        12.862544
##  6:              01             01      3.389893     0.9401115        16.697684
##  7:              01             01      2.820483     0.8857002         8.748925
##  8:              01             01      3.345492     0.9344783        15.262125
##  9:              01             01      3.843335     0.9588449        24.298324
## 10:              01             01      3.201240     0.8979416         9.798313
## 11:              01             01      3.194270     0.8805337         8.370564
## 12:              01             01      2.615324     0.8670521         7.521742
## 13:              01             01      3.515454     0.9382745        16.200749
## 14:              01             01      2.557280     0.8453968         6.468172
## 15:              01             01      3.929186     0.9397792        16.605561
##     Value_sum Value_count Value_describe_vars Value_describe_n
##  1:    244879        2284                   1             2284
##  2:     81588        1080                   1             1080
##  3:    274792        2477                   1             2477
##  4:    104195        2269                   1             2269
##  5:    124676        1465                   1             1465
##  6:    180497        1786                   1             1786
##  7:    296246        2255                   1             2255
##  8:    177010        1538                   1             1538
##  9:     59966        2644                   1             2644
## 10:    303345        2993                   1             2993
## 11:     95971        2636                   1             2636
## 12:    343490        2223                   1             2223
## 13:    149100        2386                   1             2386
## 14:    277048        1817                   1             1817
## 15:     64118        3923                   1             3923
##     Value_describe_mean Value_describe_sd Value_describe_median
##  1:           107.21497         1521.7649                     1
##  2:            75.54444          556.4600                     1
##  3:           110.93742         1852.2801                     1
##  4:            45.92111          509.2056                     1
##  5:            85.10307          904.5528                     1
##  6:           101.06215         1040.5982                     1
##  7:           131.37295         2105.4943                     1
##  8:           115.09103         1149.9733                     1
##  9:            22.68003          235.5394                     1
## 10:           101.35149         1768.7577                     1
## 11:            36.40781          645.1812                     1
## 12:           154.51642         2652.4479                     1
## 13:            62.48952          755.9385                     1
## 14:           152.47551         2551.7136                     1
## 15:            16.34412          250.7138                     1
##     Value_describe_trimmed Value_describe_mad Value_describe_min
##  1:               1.160284                  0                  1
##  2:               1.082176                  0                  1
##  3:               1.234493                  0                  1
##  4:               1.068795                  0                  1
##  5:               1.083546                  0                  1
##  6:               1.080420                  0                  1
##  7:               1.248199                  0                  1
##  8:               1.065747                  0                  1
##  9:               1.086484                  0                  1
## 10:               1.208768                  0                  1
## 11:               1.136019                  0                  1
## 12:               1.295110                  0                  1
## 13:               1.115707                  0                  1
## 14:               1.252234                  0                  1
## 15:               1.083147                  0                  1
##     Value_describe_max Value_describe_range Value_describe_skew
##  1:              48411                48410            21.84543
##  2:              10411                10410            11.49921
##  3:              68661                68660            29.32449
##  4:              18252                18251            24.92426
##  5:              19865                19864            17.48608
##  6:              26055                26054            16.62791
##  7:              70946                70945            25.02978
##  8:              32536                32535            19.00613
##  9:               5379                 5378            15.44781
## 10:              80197                80196            35.45585
## 11:              28170                28169            35.63449
## 12:              81381                81380            23.99526
## 13:              27428                27427            24.99102
## 14:              83741                83740            26.01411
## 15:              10463                10462            29.78536
##     Value_describe_kurtosis Value_describe_se distinct_barcodes
##  1:                564.8589         31.841939              2284
##  2:                159.6777         16.932540              1080
##  3:                969.0394         37.217196              2477
##  4:                788.0556         10.689956              2269
##  5:                342.6372         23.632797              1465
##  6:                330.3889         24.623077              1786
##  7:                716.3839         44.338480              2255
##  8:                457.8748         29.323081              1538
##  9:                276.7000          4.580710              2644
## 10:               1478.2344         32.330691              2993
## 11:               1447.9758         12.566345              2636
## 12:                625.3701         56.257072              2223
## 13:                793.6929         15.475733              2386
## 14:                757.2388         59.862447              1817
## 15:               1045.4960          4.002848              3923
```

```
desc_stats %>%
  readr::write_tsv(file = "descriptive_stats_by_ID.tsv")

# Aggregation and new stats ----
agg_key <- c("SubjectID")
agg <- aggregate_values_by_key(matrix, af, key = agg_key,
                               group = "BarcodeSeq",
                               join_af_by = pcr_id_column())
agg
```

```
## # A tibble: 33,267 × 3
##    BarcodeSeq             SubjectID Value_sum
##    <chr>                  <chr>         <int>
##  1 AAAAAAAACACGGAGAACGACG C3                2
##  2 AAAAAAAACGCGAACAACTACG C3                1
##  3 AAAAAAAACTCAAAAAAGAAAT C3                1
##  4 AAAAAAAATTTACACAAAGAAA A4                1
##  5 AAAAAAAATTTTTAAACGTACC A0                1
##  6 AAAAAACATATCTATAGTTACC A0                1
##  7 AAAAAAGACGACGATAGGCACG C1                1
##  8 AAAAAAGACGTTTATAGGTGTA A2                1
##  9 AAAAAAGACTGCGACAAAAGGG A4                1
## 10 AAAAAAGACTTTGATAACCACG C3                1
## # … with 33,257 more rows
```

```
agg %>%
  readr::write_tsv(file = "aggregate_matrix.tsv")


agg_meta_functions <- tibble::tribble(
  ~Column, ~Function, ~Args, ~Output_colname,
  "TimePoint", ~mean(.x, na.rm = TRUE), NA, "{.col}_avg",
  "CellMarker", ~length(unique(.x)), NA, "distinct_cell_marker_count",
  "ID", ~length(unique(.x)), NA, "distinct_id_count"
)
agg_meta <- aggregate_metadata(
  af, aggregating_functions = agg_meta_functions,
  grouping_keys = agg_key
)
agg_meta
```

```
## # A tibble: 9 × 4
##   SubjectID TimePoint_avg distinct_cell_marker_count distinct_id_count
##   <chr>             <dbl>                      <int>             <int>
## 1 A0                   21                          1                 2
## 2 A1                   21                          1                 2
## 3 A2                   21                          1                 1
## 4 A3                   21                          1                 1
## 5 A4                   21                          1                 2
## 6 C0                   21                          1                 1
## 7 C1                   21                          1                 2
## 8 C2                   21                          1                 2
## 9 C3                   21                          1                 2
```

```
agg_meta %>%
  readr::write_tsv(file = "aggregate_metadata.tsv")

agg_stats <- sample_statistics(agg, agg_meta,
                               sample_key = agg_key,
                               value_columns = "Value_sum")$metadata %>%
  dplyr::rename(distinct_barcodes = "nIS")
agg_stats
```

```
## # A tibble: 9 × 23
##   SubjectID TimePoint_…¹ disti…² disti…³ Value…⁴ Value…⁵ Value…⁶ Value…⁷ Value…⁸
##   <chr>            <dbl>   <int>   <int>   <dbl>   <dbl>   <dbl>   <int>   <int>
## 1 A0                  21       1       2    3.24   0.929   14.1   326467    3304
## 2 A1                  21       1       2    3.47   0.927   13.7   378987    4631
## 3 A2                  21       1       1    3.18   0.922   12.9   124676    1465
## 4 A3                  21       1       1    3.39   0.940   16.7   180497    1786
## 5 A4                  21       1       2    3.29   0.930   14.3   473256    3718
## 6 C0                  21       1       1    3.84   0.959   24.3    59966    2644
## 7 C1                  21       1       2    3.39   0.920   12.6   399316    5538
## 8 C2                  21       1       2    3.05   0.903   10.3   492590    4526
## 9 C3                  21       1       2    3.00   0.886    8.77  341166    5655
## # … with 14 more variables: Value_sum_describe_vars <dbl>,
## #   Value_sum_describe_n <dbl>, Value_sum_describe_mean <dbl>,
## #   Value_sum_describe_sd <dbl>, Value_sum_describe_median <dbl>,
## #   Value_sum_describe_trimmed <dbl>, Value_sum_describe_mad <dbl>,
## #   Value_sum_describe_min <dbl>, Value_sum_describe_max <dbl>,
## #   Value_sum_describe_range <dbl>, Value_sum_describe_skew <dbl>,
## #   Value_sum_describe_kurtosis <dbl>, Value_sum_describe_se <dbl>, …
```

```
agg_meta %>%
  readr::write_tsv(file = "aggregate_descriptive_stats.tsv")

# Abundance ----
abundance <- compute_abundance(agg, columns = "Value_sum", key = agg_key)
abundance
```

```
## # A tibble: 33,267 × 5
##    BarcodeSeq             SubjectID Value_sum Value_sum_RelAbundance Value_sum…¹
##    <chr>                  <chr>         <int>                  <dbl>       <dbl>
##  1 AAAAAAAACACGGAGAACGACG C3                2             0.00000586    0.000586
##  2 AAAAAAAACGCGAACAACTACG C3                1             0.00000293    0.000293
##  3 AAAAAAAACTCAAAAAAGAAAT C3                1             0.00000293    0.000293
##  4 AAAAAAAATTTACACAAAGAAA A4                1             0.00000211    0.000211
##  5 AAAAAAAATTTTTAAACGTACC A0                1             0.00000306    0.000306
##  6 AAAAAACATATCTATAGTTACC A0                1             0.00000306    0.000306
##  7 AAAAAAGACGACGATAGGCACG C1                1             0.00000250    0.000250
##  8 AAAAAAGACGTTTATAGGTGTA A2                1             0.00000802    0.000802
##  9 AAAAAAGACTGCGACAAAAGGG A4                1             0.00000211    0.000211
## 10 AAAAAAGACTTTGATAACCACG C3                1             0.00000293    0.000293
## # … with 33,257 more rows, and abbreviated variable name
## #   ¹​Value_sum_PercAbundance
```

```
abundance %>%
  readr::write_tsv(file = "abundance_by_subject.tsv")

sessionInfo()
```

```
## R version 4.2.1 (2022-06-23)
## Platform: x86_64-apple-darwin17.0 (64-bit)
## Running under: macOS Big Sur ... 10.16
## 
## Matrix products: default
## BLAS:   /Library/Frameworks/R.framework/Versions/4.2/Resources/lib/libRblas.0.dylib
## LAPACK: /Library/Frameworks/R.framework/Versions/4.2/Resources/lib/libRlapack.dylib
## 
## locale:
## [1] en_US.UTF-8/en_US.UTF-8/en_US.UTF-8/C/en_US.UTF-8/en_US.UTF-8
## 
## attached base packages:
## [1] stats     graphics  grDevices utils     datasets  methods   base     
## 
## other attached packages:
## [1] ISAnalytics_1.7.6 magrittr_2.0.3   
## 
## loaded via a namespace (and not attached):
##  [1] sass_0.4.2           tidyr_1.2.1          bit64_4.0.5         
##  [4] vroom_1.6.0          jsonlite_1.8.2       splines_4.2.1       
##  [7] bslib_0.4.0          shiny_1.7.2          highr_0.9           
## [10] cellranger_1.1.0     yaml_2.3.5           pillar_1.8.1        
## [13] lattice_0.20-45      glue_1.6.2           digest_0.6.29       
## [16] promises_1.2.0.1     colorspace_2.0-3     psych_2.2.9         
## [19] Matrix_1.5-1         htmltools_0.5.3      httpuv_1.6.6        
## [22] pkgconfig_2.0.3      haven_2.5.1          purrr_0.3.4         
## [25] xtable_1.8-4         scales_1.2.1         openxlsx_4.2.5      
## [28] later_1.3.0          rio_0.5.29           tzdb_0.3.0          
## [31] BiocParallel_1.31.12 tibble_3.1.8         mgcv_1.8-40         
## [34] generics_0.1.3       ggplot2_3.3.6        ellipsis_0.3.2      
## [37] cachem_1.0.6         datamods_1.3.4       mnormt_2.1.1        
## [40] cli_3.4.1            Rcapture_1.4-4       crayon_1.5.2        
## [43] readxl_1.4.1         mime_0.12            evaluate_0.16       
## [46] fs_1.5.2             fansi_1.0.3          nlme_3.1-159        
## [49] MASS_7.3-58.1        forcats_0.5.2        foreign_0.8-83      
## [52] vegan_2.6-2          tools_4.2.1          data.table_1.14.2   
## [55] hms_1.1.2            lifecycle_1.0.2      stringr_1.4.1       
## [58] munsell_0.5.0        cluster_2.1.4        zip_2.2.1           
## [61] compiler_4.2.1       jquerylib_0.1.4      rlang_1.0.6         
## [64] grid_4.2.1           rstudioapi_0.14      rmarkdown_2.16      
## [67] shinyWidgets_0.7.3   gtable_0.3.1         codetools_0.2-18    
## [70] curl_4.3.2           R6_2.5.1             lubridate_1.8.0     
## [73] knitr_1.40           dplyr_1.0.10         fastmap_1.1.0       
## [76] bit_4.0.4            utf8_1.2.2           permute_0.9-7       
## [79] readr_2.1.3          stringi_1.7.8        parallel_4.2.1      
## [82] Rcpp_1.0.9           vctrs_0.4.2          tidyselect_1.1.2    
## [85] xfun_0.33
```
